# Integrating Mortality Risk and the Adaptiveness of Hibernation

**DOI:** 10.3389/fphys.2020.00706

**Published:** 2020-07-10

**Authors:** Théo Constant, Sylvain Giroud, Vincent A. Viblanc, Mathilde L. Tissier, Patrick Bergeron, F. Stephen Dobson, Caroline Habold

**Affiliations:** ^1^UMR 7178, Centre National de la Recherche Scientifique, Institut Pluridisciplinaire Hubert CURIEN, Université de Strasbourg, Strasbourg, France; ^2^Department of Interdisciplinary Life Sciences, Research Institute of Wildlife Ecology, University of Veterinary Medicine Vienna, Vienna, Austria; ^3^Department of Biological Sciences, Bishop’s University, Sherbrooke, QC, Canada; ^4^Department of Biological Sciences, Auburn University, Auburn, AL, United States

**Keywords:** activity patterns, hibernation, life history, temporal organization of activity, time and energy allocations, trade-off, longevity, mortality risk

## Abstract

Low mortality rate is often associated with slow life history, and so far, has mainly been assessed through examinations of specific adaptations and lifestyles that limit mortality risk. However, the organization of activity time budgets also needs to be considered, since some activities and the time afforded for performing them may expose animals to higher mortality risks such as increased predation and/or increased metabolic stress. We examined the extent of activity time budgets contribution to explaining variation in life history traits in mammals. We specifically focused on hibernating species because of their marked seasonal cycle of activity/inactivity associated with very different mortality risks. Hibernation is considered a seasonal adaptation to prolonged periods of food shortage and cold. This inactivity period may also reduce both extrinsic and intrinsic mortality risks, by decreasing exposure to predators and drastically reducing metabolic rate. In turn, reduction in mortality may explain why hibernators have slower life history traits than non-hibernators of the same size. Using phylogenetically controlled models, we tested the hypothesis that longevity was positively correlated with the hibernation season duration (the time spent between immergence and emergence from the hibernaculum or den) across 82 different mammalian species. We found that longevity increased significantly with hibernation season duration, an effect that was particularly strong in small hibernators (<1.5 kg) especially for bats. These results confirm that hibernation not only allows mammals to survive periods of energy scarcity, but further suggest that activity time budgets may be selected to reduce mortality risks according to life history pace.

## Introduction

Energy is the fundamental requirement for life. Its acquisition, storage, and metabolic use shape the diversity of lifestyles in all living organisms (Brown et al., 2004). Because energy availability to organisms is limited under natural conditions, in terms of its acquisition in time and space, as well as its quantity and quality, organisms have to simultaneously maximize investments into all biological functions, and to compromise the allocation of metabolic energy among competing demands (Lack, 1966; Williams, 1966; Hirshfield and Tinkle, 1975; Reznick, 1985). For example, demographic characteristics may affect energy investments among biological traits such as growth, reproduction, and somatic maintenance. These trade-offs have led to the evolution of specific life history strategies (Stearns, 1992).

Attributes of life histories generally scale with body size such that large animal species usually take longer to develop and mature, have lower annual reproductive rates, and live longer compared to small species (Blueweiss et al., 1978; Speakman, 2005). However, life history variations that are specific to a particular body size are also observed along a fast-slow continuum (Oli, 2004; Bielby et al., 2007; Dobson and Oli, 2007; Jones et al., 2008; Jeschke and Kokko, 2009). For a given body size, most species trade off investments between reproduction and self-maintenance. For example, a species with fast life history strategy will exhibit faster growth, earlier reproduction, larger annual reproductive investment, and reduced maximum life span compared to a species with a slow life history strategy, which will typically promote self-maintenance and survival over reproduction (but see Bielby et al., 2007; Jeschke and Kokko, 2009).

The evolution of the fast-slow continuum in life history strategies appears to be contingent upon individual mortality risk (Promislow and Harvey, 1990; Martin, 2015; Healy et al., 2019). Individual mortality can be due to either intrinsic (wear-and-tear of the body) or extrinsic factors, such as predation, disease, or environmental hazards. Species with slow life histories typically exhibit adaptations that limit both sources of mortality (Holmes and Austad, 1994; Wilkinson and South, 2002; Blanco and Sherman, 2005; Sibly and Brown, 2007; Munshi-South and Wilkinson, 2010; Shattuck and Williams, 2010; Turbill et al., 2011; Lewis et al., 2013; Healy et al., 2014; Healy, 2015; MacRae et al., 2015; Wu and Storey, 2016). Besides these molecular (such as oxidative stress tolerance), physiological/anatomical (such as chemical protection, horns and antlers) or lifestyle (such as arboreality) adaptations that reduce mortality risk, the organization of activity time budgets should be particularly important in shaping the variety of life histories observed in the wild. Mortality rates may change depending on the time allocated to each activity, resulting in trade-offs for which both time and energy can be optimized. For instance, activities that contribute the most to reproductive success are often energy-intensive (Alonso-Alvarez et al., 2004) and associated with higher risks of extrinsic mortality (Magnhagen, 1991). However, the temporal dimension of energy allocation trade-offs in relation to the evolution of life history strategies has been little examined (see Healy et al., 2014).

Here, we first examined the extent to which patterns of relative activity and inactivity might explain variation in life history traits in mammals. We specifically focused on hibernating species because of their marked seasonal cycle of activity/inactivity, which is associated with very different risks of mortality. From an intrinsic perspective, hibernation is a period of metabolic depression where energy requirements are reduced to minimal levels compared to the active season (Ruf and Geiser, 2015). It has been suggested that energy restriction slows down the aging process (Walford and Spindler, 1997; Masoro, 2006) and is associated with enhancement of somatic maintenance (Shanley and Kirkwood, 2000). From an extrinsic perspective, even if mortality during hibernation occurs, hibernating mammals are usually hidden in burrows or shelters, which may reduce risks of predation, infections or injuries for several continuous months. As a result, hibernating species generally exhibit lower rates of mortality than similar-sized non-hibernating species during part of the year, resulting in slower life history strategies (Wilkinson and South, 2002; Turbill et al., 2011). However, previous studies linking hibernation to longevity considered hibernation as a binary trait (if animals hibernate or not), rather than a continuous adaptive response (hibernation season duration) allowing animals to restrict their period of activity during parts of the year. Yet, early data from captivity highlight a positive correlation between longevity and hibernation duration in Turkish hamsters (*Mesocricetus brandti*) (Lyman et al., 1981). In addition, a strong negative effect of mean annual temperature on hibernation season duration and annual survival rate, which is highly correlated with longevity (Turbill et al., 2011), has been shown among populations of hibernating rodent species (Turbill and Prior, 2016). This raises the question of whether, across hibernating mammals, the time spent being inactive (hibernation season duration) influences maximal longevity, a key feature characteristic of fast and slow life history strategies.

In the present study, we tested for a positive association between the hibernation season duration and longevity across 82 mammalian species. For this examination, we tested for effect of body size on longevity while controlling statistically for phylogeny. If indeed hibernation season duration is part of a strategy that minimizes mortality, we predicted that a longer hibernation season duration should be positively associated with species maximum longevity, especially in small mammals (<1.5 kg) that exhibit greater longevity than non-hibernators of the same size (Turbill et al., 2011). In addition, if metabolic reduction during hibernation slows aging (Lyman et al., 1981; Turbill et al., 2013; Wu and Storey, 2016), we predicted that the effect of hibernation season duration on longevity should increase with the percentage of metabolic reduction during hibernation compared to euthermia.

## Materials and Methods

### Review Criteria

We conducted the review using the search engine Google Scholar^1^ and considered articles up to and including December 2019. We based our survey on the hibernating species listed in Turbill et al. (2011) and Ruf and Geiser (2015), and further identified nine other species, mainly ground squirrels and bats, not mentioned in any of the lists. All the 152 hibernators that were examined in this process are summarized in Supplementary Material (see Supplementary Table S1). We excluded species for which hibernation was restricted to only a few populations or under specific conditions, namely two species, the black-tailed prairie dog (*Cynomys ludovicianus*; Gummer, 2005; Lehmer et al., 2006) and the polar bear (*Ursus maritimus*; Amstrup and DeMaster, 2003). We did not include non-seasonal hibernating species capable of entering multi-day torpors at any time of the year. Indeed, for such cases, it is difficult to accurately measure the time spent in hibernation over a year, which may also vary considerably among individuals and between years. Thus, we excluded hibernating elephant shrew species (*Elephantulus* sp.) and hibernating marsupials with the exception of the mountain pygmy possum (*Burramys parvus*), reported to be a seasonal hibernator (Lovegrove et al., 2001; Geiser and Körtner, 2010).

### Longevity and Body Mass Data

Data on maximum longevity, hereafter referred as longevity, and average body mass for the list of hibernating species previously identified were mainly obtained from the AnAge data base (The Animal Aging and Longevity Database^2^; Human Ageing and Genomic Resources; Magalhães and Costa, 2009), and complemented these data with information from the PanTHERIA data base (Ecological Society of America^3^; Jones et al., 2009) from two reviews on mammalian longevity (Heppell et al., 2000; Wilkinson and South, 2002) and from a specific search in Google Scholar combining the following terms: “longevity” OR “life history” AND scientific or common names of species. For this specific search in Google Scholar, we considered both old and new nomenclatures for ground squirrel species, and only selected long-term field studies since they provide a good estimate of maximum longevity. Moreover, we specifically investigated the source of the data (captive vs. wild) and only retained longevity data where the source was available, to control for captivity effects on longevity (see Supplementary Table S2 for references).

We determined the arboreality lifestyle of the species in order to statistically control (see below section “Statistics”) for higher longevity (Kamilar et al., 2010; Shattuck and Williams, 2010; Healy et al., 2014). For this, we conducted a review of peer-reviewed scientific journal Mammalian Species^4^ and used Google Scholar. The search criteria were based on combining the following terms: “arboreal” OR “semi-arboreal” OR “climb tree” AND scientific or common names of species. We completed our search by examining the mammalian lifestyle databases of the following articles (Healy et al., 2014; Hidasi-Neto et al., 2015). Our arboreality factor included 16 arboreal and semi-arboreal species, which feed, nest, or escape from predators, at least frequently, by climbing into trees (see Supplementary Table S2).

### Hibernation Season Duration Data

#### Reviewing Strategy

Relatively few studies have investigated hibernation duration with body temperature recorders on wild mammals. However, several studies have estimated hibernation season duration from capture-mark-recapture records or direct observations, thus assessing periods of inactivity. Although for some species, the duration of hibernation measured as the period between the first and last torpor bout is probably shorter than the period of inactivity (Young, 1990; Williams et al., 2014; Siutz et al., 2016), these measures should still provide reasonable estimates of the duration of energy savings, thereby allowing species to be compared with each other.

We reviewed the literature to retrieve estimates of the hibernation season duration as the time (in days) spent between immergence and emergence from the hibernaculum or den (with little or no movement outside). The search criteria were based on combining the following terms: “hibernation” OR “hibernation duration” OR “denning” (exclusively for bears) OR “roosting” (exclusively for bats) AND scientific or common names of species. In order to minimize heterogeneity in the scales at which the data were measured (e.g., individual, population), we considered maximum hibernation season duration obtained from same-sex adult groups, either male or female depending on the species (and recorded maximum hibernation season duration from overall population data when more precise data were unavailable).

#### Inclusion and Exclusion Criteria

We prioritized studies for which the methodology for estimating hibernation season duration was described (capture-mark-recapture, direct observation and body temperature recording). These criteria included 64 species. We also included studies based on road kills as an index of activity/inactivity. This criterion has already been used to evaluate changes in hedgehog abundance (Morris and Morris, 1988; Bright et al., 2015; Wembridge et al., 2016) and was used for 2 species in our data set [The Algerian hedgehog (*Atelerix algirus*) and the Southern white-breasted hedgehog (*Erinaceus concolor*)]. Finally, in cases where the above criteria were not available, we included studies for which a precise hibernation period was mentioned but the methodology could not be assessed. This criterion included 16 species (see Supplementary Table S2, labeled species).

We excluded studies for which periods of extreme inactivity were measured only once in a population. This criterion excluded three maximum hibernation season data: 8 months for the little pocket mouse (*Perognathus longimembris*; Kenagy and Bartholomew, 1985); 6 months for the long-tailed pocket mouse (*Chaetodipus formosus*; Kenagy and Bartholomew, 1985); and 11 months for the Eastern chipmunk (*Tamias striatus*; Munro et al., 2008).

The availability or absence of data (longevity and hibernation season duration) in the literature for the 152 species examined is specified in Supplementary Material (see Supplementary Table S1). In total, our literature search allowed inclusion of 82 hibernating mammals in the analyses including 80 placental mammals, 27 bats, one marsupial (the mountain pygmy possum), and one monotreme [the short-beaked echidna (*Tachyglossus aculeatus*)]. Longevity, body mass and hibernation season duration data for these species are available in Supplementary Material (see Supplementary Table S2).

### Phylogenetic Data

We downloaded 100 phylogenetic mammalian trees^5^ (see (Upham et al., 2019), focusing on the 82 species in our data set (see Supplementary Table S2). These trees were used to construct strict consensus trees for the hibernating species of our study, where the included clades were those present in all the 100 phylogenetic mammalian trees (Paradis, 2011). Because we ran subsequent analyses on different subsets of the global dataset (see section “Phylogenetic Data” below), we constructed separate phylogenetic trees on (1) the full hibernator data set (*N* = 82 species), (2) the data set excluding bats (*N* = 55 species), (3) the data set with only deep hibernators (see below) excluding bats (*N* = 46 species), and (4) the data set excluding bats and hibernators >1.5 kg (*N* = 44 species) (see Supplementary Figure S1).

The rationale for eliminating bats from some analyses was to compare the specific effect of hibernation season duration on longevity between bats which have very distinct characteristics (i.e., flight capacity, highly gregarious behavior during hibernation, Austad and Fischer, 1991) and other hibernators.

The metabolic rate during torpor is not known for all hibernating species studied (Ruf and Geiser, 2015). To test the prediction that the effect of hibernation season duration on longevity should increase with metabolic reduction, we compared the effect of hibernation season duration on the longevity of two groups including (all hibernators excluding bats) or excluding (only deep hibernators without bats) species reducing their energy expenditure during hibernation by less than 90% compared to the euthermic state. These comprise *Ursidae* species and the European badger (*Meles meles*) that reduce their total energy expenditure from 33 to 75% during hibernation compared to the euthermic state (Hellgren, 1998; Watts and Jonkel, 1988; Tøien et al., 2011; Ruf and Geiser, 2015) and small tropical hibernators such as *Cheirogaleidae* and *Tenrecidae* species, which show a 70% reduction (Dausmann et al., 2009; Wein, 2010). Thus, the “deep hibernator” group includes the species capable of reducing their total energy expenditure by about 90% or more during hibernation as compared to the euthermic state and reaching a body temperature during torpor below 10°C (mainly small Holarctic species; Heldmaier et al., 2004).

In addition, the analyses of Turbill et al., 2011 indicated a body mass threshold of 1.5 kg, below which the benefits of hibernation (compared to non-hibernation) for longevity increased. To test the effect of hibernation season duration on longevity between hibernators <1.5 kg and larger ones, we used the data set excluding bats and hibernators >1.5 kg.

Branch lengths for respective consensus trees were calculated with the “compute.brlen” function from the “ape” package based on Grafen’s (1989) computations, and were used to compute PGLS models with the “caper” package in R (see section “Phylogenetic Data” below).

### Statistics

We tested for a significant relationship between hibernation season duration and species maximum longevity, using phylogenetic generalized least squares (PGLS) models with the “ape 5.0,” “apTreeshape 1.5,” and “caper 1.0” packages in R v. 3.6.2 (Paradis, 2011; Orme et al., 2013; Paradis and Schliep, 2019; R Core Team, 2019). We thus statistically “controlled” for the influence of the phylogenetic relationships among species on the variables before evaluating relationships. In addition, the relative effect of the phylogenetic tree on the linear model could be estimated as a λ parameter, ranging between 0 (covariation among species measurements is independent of co-ancestry) and 1 (covariance entirely explained by co-ancestry). Testing the models with λ = 0 allowed comparison to λ-positive models, and thus the extent to which phylogeny influenced analyses of the models examined. In addition to phylogeny, our model evaluated the influences of average body mass (of adults) of the different species, bats and arboreality lifestyle, and the fact that some data were acquired from captive and wild populations (see below).

We ran PGLS models for the four different conditions listed above (Table 1). Longevity was the dependent variable in all our models, and hibernation season duration and species average body mass were independent variables. In all models, body mass and longevity were log-transformed to normalize their distributions, and all independent variables were standardized (using *z-scores*), so that their coefficients are directly comparable as estimates of effect sizes (Abdi, 2007). In the original models, we included the interaction *hibernation season duration x body mass* to test for the possibility that the effect of hibernation season duration on longevity was more important for species of small body mass (see Figure 2 in Turbill et al., 2011), as well as a “captive/wild” factor to account for captivity-related variation in longevity (Tidière et al., 2016). However, these factors were parsimoniously dropped in the final models based on Akaike’s Information Criterion (AIC). Among the models within ΔAIC < 2 (ΔAIC_i_ = AIC_i_-AIC_min_), we kept the model with the lowest number of terms (see Supplementary Table S3). Nevertheless, body mass was retained throughout our models, because of the dominance that it shows as a primary axis of energetics and life history (Stearns, 1992; Brown et al., 2004). In order to control for higher longevity due to particular lifestyles, we added a “bat (yes/no)” factor in the full model (Wilkinson and South, 2002; Turbill et al., 2011) and an “arboreality (yes/no)” factor in each model (Kamilar et al., 2010; Shattuck and Williams, 2010; Healy et al., 2014). We limited the number of additional predicators in order to maintain sufficient statistical power with respect to the sample size (Table 2; Mundry, 2014).

**TABLE 1:** Summary of models and datasets.

**Model**	**1**	**2**	**3**	**4**	**5**
**Model type**	**PGLS**	**PGLS**	**PGLS**	**PGLS**	**Linear model**
Hibernators > 1.5 kg	x	x			
Hibernators < 1.5 kg	x	x		x	
Deep hibernators	x	x	x		
Bats	x				x
Sample size	82	55	46	44	27
Arboreal and semi-arboreal species	16	16	11	15	0
Hibernation season duration range (day)	105−296	105−296	105−296	105−296	120−255
Longevity range (year)	3.5−49.5	3.5−49.5	3.5−49.5	3.5−29	6−41
Body mass range (g)	4.6−227500	8−227500	8−7300	8−958	4.6−28.55

*The category known as “deep hibernator” includes the species capable of reducing their total energy expenditure by about 90% or more during hibernation as compared to the euthermic state and reaching a body temperature during torpor below 10°C (mainly small Holarctic species; Heldmaier et al., 2004). Crosses indicate group(s) included in each model.*

**TABLE 2:** Regression results for the best models explaining variation in longevity among hibernators species.

			**λ_ML_**				**λ = 0**		
			**λ_ML_**	**β ± SE**	***t*-value**	***p*-value**	**β ± SE**	***t*-value**	***p*-value**
Phylogenetic correction (PGLS)	Model 1: All hibernators (82 species)	Intercept z-Hibernation duration z-log (Body mass) bat arboreality	λ_ML_ = 0.736 CI_95_ = [NA-0.924]	0.986 ± 0.111	8.847	< 0.001***	0.834 ± 0.032	25.778	< 0.001***
				0.051 ± 0.020	2.484	0.015*	0.028 ± 0.020	1.343	0.183
				0.163 ± 0.043	3.800	< 0.001***	0.216 ± 0.026	8.409	< 0.001***
				0.531 ± 0.170	3.131	0.002**	0.656 ± 0.060	10.903	< 0.001***
				0.163 ± 0.075	2.175	(0.033*	0.182 ± 0.054	3.387	0.001**

	Model 2: Hibernators without bats (55 species)	Intercept z-Hibernation duration z-log (Body mass) arboreality	λ_ML_ = 0.849 CI_95_ = [0.238−0.969]	0.970 ± 0.112	8.682	< 0.001***	0.841 ± 0.031	27.439	< 0.001***
				0.058 ± 0.022	2.645	0.011*	0.004 ± 0.022	0.200	0.842
				0.171 ± 0.038	4.442	< 0.001***	0.217 ± 0.024	8.920	< 0.001***
				0.191 ± 0.070	2.738	0.008**	0.173 ± 0.051	3.409	0.001**

	Model 3: Deep hibernators without bats (46 species)	Intercept z-Hibernation duration z-log (Body mass)	λ_ML_ = 0.850 CI_95_ = [0.480-0.960]	1.025 ± 0.107	9.611	< 0.001***	0.876 ± 0.026	33.483	< 0.001***
				0.051 ± 0.022	2.279	0.028*	0.008 ± 0.023	0.342	0.734
				0.100 ± 0.054	1.833	0.074.	0.173 ± 0.034	5.120	< 0.001***

	Model 4: Small hibernators (<1.5 kg) without bats (44 species)	Intercept z-Hibernation duration z-log (Body mass) arboreality	λ_ML_ = 0.740 CI_95_ = [0.293−0.924]	0.867 ± 0.084	10.240	< 0.001***	0.820 ± 0.029	28.543	< 0.001***
				0.061 ± 0.020	2.994	0.005**	0.024 ± 0.021	1.148	0.258
				0.014 ± 0.053	0.264	0.793	0.121 ± 0.042	2.864	0.007**
				0.204 ± 0.069	2.937	0.005**	0.191 ± 0.048	4.002	< 0.001***

No phylogenetic correction (linear model)	Model 5: Bats only (27 species)	Intercept z-Hibernation duration	Not estimated	1.453 ± 0.168	8.619	< 0.001*	Not estimated		
				0.101 ± 0.045	2.215	0.036*			
				0.141 ± 0.179	0.785	0.440			
		z-log (Body mass)							

Z-standardized model estimates (ß) for the effects of hibernation season duration and body mass on species maximum longevity. The phylogenetic effect is estimated by λ_ML_. Both body mass and longevity were log-transformed before the analyses. For comparison, we have provided estimates for λ constrained to zero (no effect of phylogeny). The model for bats was a simple linear model not controlling for phylogeny, due to limited sample size in this group. In the model with all hibernators, the NA value in the confidence interval for *λ_*ML*_* indicates that the caper package could not calculate the full confidence interval. An NA value is considered as 0. Significant results are represented as *p < 0.05, **p < 0.01, ***p < 0.001.

For the final models, the level of covariation in maximum longevity among species was estimated by maximum likelihood (λ_ML_).

Within bats (individuals from wild populations only), we were not able to estimate the effects of body mass and hibernation season duration on longevity, while controlling statistically for phylogeny. We had too few species of bats (*N* = 27; Münkemüller et al., 2013) for properly evaluating the phylogenetic signal (the lower CI bound for the phylogenetic signal could not be estimated; see Supplementary Material, Supplementary Figure S2). Thus, we present simple linear regressions for this group later indicated as model 5 (Tables 1, 2).

## Results

The characteristics of the models and data used are summarized in Table 1. For each model, hibernation season duration and longevity were similar in range between the different datasets (Table 1). Naturally, the range of body mass was much smaller when considering only deep hibernators, small species and bats.

### Model 1 (*N* = 82)

**FIGURE 1:**
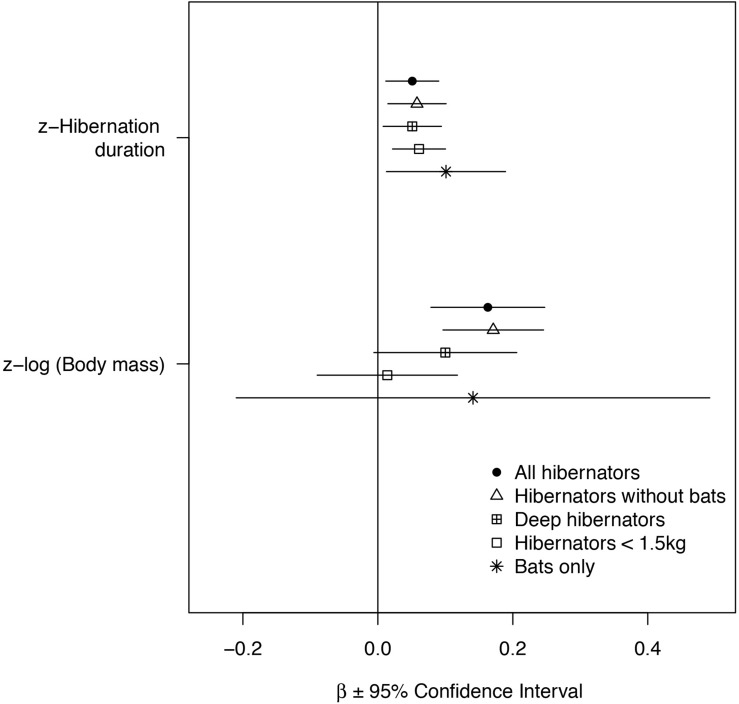
Effects of hibernation season duration and body mass on longevity. Z-standardized model coefficients are presented ±95% Confidence Interval (1.96xSE). Both body mass and longevity were log-transformed before the analyses. The effect sizes are presented for PGLS models for all mammals (*N* = 82), mammals without bats (*N* = 55), deep hibernators without bats (*n* = 46) and small mammals without bats (*N* = 44). For comparison, effect sizes from a simple linear model not accounting for phylogeny are presented for bats only (*N* = 27).

**FIGURE 2:**
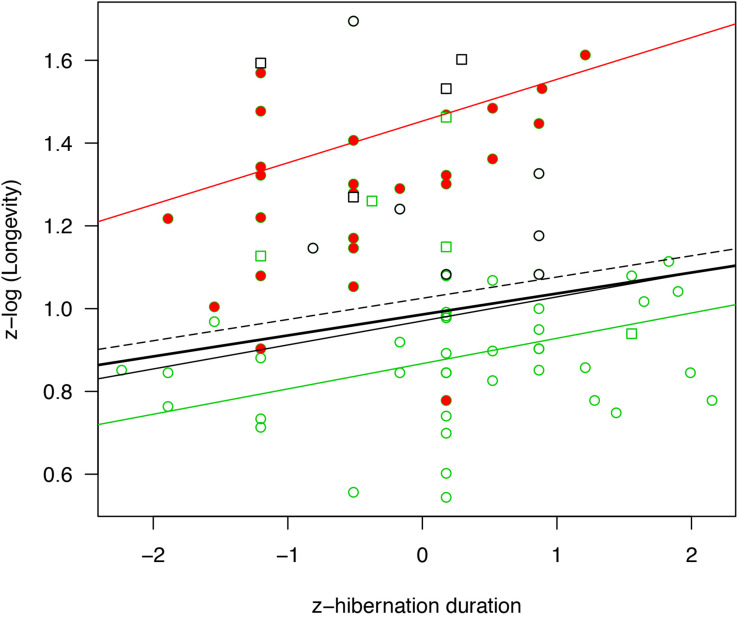
Relationship between hibernation season duration (standardized) and longevity (log-transformed and standardized). The regression lines are presented for PGLS models for all mammals (bold black line, *N* = 82, *p* = 0.015), all hibernators without bats (black line, *N* = 55, *p* = 0.011), deep hibernators without bats (dashed line, *N* = 46, *p* = 0.028) and small hibernators without bats (green line, *N* = 44, *p* = 0.005). For comparison, effect sizes from a simple linear model not accounting for phylogeny are presented for bats only (red line, *N* = 27, *p* = 0.036). Full red circles highlight bat species, the squares highlight species reducing their energy expenditure during hibernation by less than 90% compared to the euthermic state, green item highlight species below 1.5 kg, and black circle highlight all remaining hibernators above 1.5 kg in the data set. Please note that some dots referring to bat species are superimposed because the corresponding species have the same hibernation season durations and longevities.

Accounting for the effect of phylogeny, variation in longevity was positively associated with hibernation season duration and body mass across all hibernating mammals (Figures 1, 2 and Table 2). On average, bats had significantly longer lifespans (83%, $\bar{\text{x}}$ = 21 years, *SD* = 8.7, *N* = 27) as well as species with an arboreal lifestyle (12.6%, $\bar{\text{x}}$ = 12.9 years, *SD* = 9.2, *N* = 16), than other non-flying and non-arboreal mammals ($\bar{\text{x}}$ = 11.5 years, *SD* = 9.7, *N* = 39) (Table 2).

### Model 2 (*N* = 55)

Removing hibernating bats from the analyses showed that hibernation season duration still had a significant positive effect on longevity (Figures 1, 2 and Table 2). The positive effect of body mass and an arboreal lifestyle on longevity remained, mammals with a higher body mass or an arboreal lifestyle exhibiting significantly longer lifespan (Figures 1, 2 and Table 2).

### Models 3 and 4 (*N* = 46 and *N* = 44, Respectively)

When only deep hibernators (model 3) and small hibernators <1.5 kg (excluding bats) were considered (model 4), we found a positive effect of hibernation season duration on longevity (Figures 1, 2 and Table 2). The positive effect of arboreality lifestyle on longevity only remained for small hibernators (model 4).

Among the above models, the effect of hibernation season duration was slightly higher for small hibernators <1.5 kg and highly significant (Figures 1, 2 and Table 2). These models showed a strong influence of phylogeny on the results (Table 2). Ignoring the effect of phylogeny by constraining λ to 0 removed the effect of hibernation season duration on longevity (Table 2).

### Model 5 (*N* = 27)

Because our sample size for bats alone was too small, we could not perform an analysis controlling for phylogeny. Instead, we ran simple linear models to test for the effects of hibernation season duration and body mass on longevity. Here as well, hibernation season duration, but not body mass, was positively and significantly related to maximum longevity (Figures 1, 2 and Table 2). Hibernation season duration had an effect on bat longevity up to two times higher than in previous models excluding bats. This significant effect without accounting for phylogeny was probably due to a lower level of phylogenetic differences among bat species, compared to other models including up to eight different orders.

## Discussion

### Influence of Hibernation Season Duration on Longevity

Our purpose was to examine the extent to which the activity time budget explains variation in life history traits in mammals. We investigated this question in hibernating species because of their marked seasonal cycle of activity/inactivity, which is associated with very different risks of mortality (Turbill et al., 2011). While controlling for phylogeny, our study highlighted a positive influence of hibernation season duration on longevity in mammalian hibernators. These results were in agreement with an early study that tested the effect of hibernation duration on longevity in captive Turkish hamsters (Lyman et al., 1981). In agreement with Dobson (2007) and Sibly and Brown (2007), the two major axes of life histories of mammalian hibernators are body mass and lifestyle, with lifestyle contributing to the slow-fast continuum. The bat lifestyle (e.g., aerial) had the greatest influence on longevity, with a positive effect on longevity that was three times greater than the arboreality lifestyle. The effect of hibernation season duration, in addition to these lifestyles, appeared to be roughly one-third of the effect of body mass on longevity (see model estimates in Table 2).

Our results show that the effects of hibernation season duration on longevity were consistent across a wide range of body sizes, and became stronger with the limitation in body mass to small species (species <1.5 kg), especially for bats (Figure 1). Interestingly, hibernation season duration appeared to be more important than body mass in explaining longevity in the latter species. These results support the idea that hibernation (1) is an efficient strategy that limits mortality in periods of energy scarcity for some larger species facing strong energy constraints during part of the year (e.g., *Marmota* species), and (2) may be an especially effective strategy for small mammals that are expected to suffer from both higher predation rate (Cohen et al., 1993; Sinclair et al., 2003) and increased loss of energy expenditure during winter (Ruf and Geiser, 2015). In either case, increased hibernation season duration may increase both annual survival rates (Turbill and Prior, 2016) and overall longevity (this study). Note that in some cases, the lack of relationship between body mass and longevity in our study could also be due to a smaller range of body mass variation than reported in other studies (for instance in bats; Wilkinson and South, 2002).

In our study, the effect of hibernation season duration on the longevity for bats was twice that of small non-flying terrestrial mammals, though this result should be considered with caution since we were not able to control for phylogeny when considering only bats (Figure 2 and Table 2). Bats stand somewhat apart from other mammals, distinguished notably by their ability for sustained flight, an important lifestyle characteristic (Sibly and Brown, 2007). Flying is an energy-intensive activity, considerably more than terrestrial locomotion (Tucker, 1968; Thomas and Suthers, 1972). Thomas and Suthers (1972) estimated that the greater spear-nosed bat (*Phyllostomus hastatus*) increases its resting metabolic rate more than 34 times during flight, while rodents of similar size increase it only by 8-fold during terrestrial locomotion. Thus, in bats the reduction of metabolism during hibernation is particularly important compared to their period of activity (Wilkinson and South, 2002).

The marked effect of hibernation season duration on longevity in bats may also be explained by some extreme physiological adaptations to hibernation having evolved in response to specific ecological and anatomical constraints (Willis, 2017). For instance, several bat species are capable of very long torpor bouts (up to 60 days; reviewed in Ruf and Geiser, 2015), perhaps in response to their limited accumulation of internal or external energy reserves (Willis, 2017). In addition, the little brown bat (*Myotis lucifugus*), for instance, is capable of performing “heterothermic arousals,” corresponding to shallow torpor bouts (T_skin_ > 20°C), during arousal phases, and thus reduce the cost of euthermia (Jonasson and Willis, 2012; Czenze et al., 2017). This particular adaptation may be present in other bat species as well. Finally, bats are highly gregarious during hibernation and can cluster in colonies of up to thousands of individuals (Clawson et al., 1980). Huddling could enable them to reduce energy costs and water loss during hibernation, making hibernation a particularly profitable strategy (Boyles et al., 2008; Gilbert et al., 2010; Boratyński et al., 2012, 2015).

Comparisons between models either including (model 2) or excluding (model 3) species with the lowest metabolic reductions during hibernation did not reveal significant differences. These results suggest that the effect of hibernation season duration on longevity remains consistent whatever the rate of metabolic reduction reached during hibernation compared to the active state (between 70 and 90%). This finding should pave the way for future studies to specifically test this effect of metabolic reduction during hibernation.

Interestingly, in all PGLS models, removing the effect of phylogeny by constraining λ to 0 also removed the effect of hibernation season duration on longevity. This suggests that the effect of hibernation season duration on longevity is masked by the phylogenetic pattern. Thus, hibernation season duration might be a stronger explanation of variation within species or between closely related species, as shown in Turkish hamsters (Lyman et al., 1981). For instance, studies comparing populations of golden-mantled ground squirrels (*Callospermophilus lateralis*) and Columbian ground squirrels (*Urocitellus columbianus*) living along an altitudinal gradient show that populations with longer hibernation season duration generally have higher annual survival and longevity (Bronson, 1979; Murie and Harris, 1982; Dobson and Murie, 1987).

Although our results highlight an association between hibernation season duration and longevity, they do not provide a causal mechanism through which such an association might arise. Periods of prolonged inactivity are likely to increase longevity through the integration of multiple factors affecting both intrinsic and extrinsic mortality, as discussed below.

### Factors Affecting Extrinsic and Intrinsic Mortality and the Evolution of Hibernation

Energetic stress, when energy demand is greater than energy availability in the environment, has a proximate role in the regulation of hibernation pattern (Vuarin and Henry, 2014). However, few studies have focused on the causal link between energetic stress, and the timing of hibernation immergence and emergence (e.g., Humphries et al., 2002). Thus, the hypothesis that hibernation occurs primarily as a response to an energetic stress has not been completely studied (focusing on torpor bouts frequency, depth and duration). To the best of our knowledge, the only study having measured both energy availability in the environment and individual energy expenditure before immergence in hibernation shows in eastern chipmunks (*Tamias striatus*), that immergence occurs while food is still plentiful and climatic conditions are still favorable for maximizing energy storage (Humphries et al., 2002). Other observations also suggest that immergence into hibernation while food is still available seems common, at least in sciurids (Humphries et al., 2003) and for the little pocket mouse (Barnes and Carey, 2004). Thus, food availability and ambient temperature alone may not be sufficient to explain the phenology of immergence.

Other evidence suggests that hibernation is not initiated solely in response to deficiencies in energy, water, or poor food quality. Many observations suggest that early immergence (before energetically stressful periods start) occurs when the benefits of reproduction are low. For instance, in years of low beech seed abundance, the edible dormouse (*Glis glis*) skips reproduction, quickly accumulates fat reserves, and is able to hibernate for up to 11 months (Hoelzl et al., 2015). This occurs even if food in the environment is sufficient to allow the edible dormouse to remain active but not to reproduce. Similarly, Eastern chipmunks skip reproduction and cease foraging for almost a full year when food availability is particularly low (Munro et al., 2008). It seems that at that time, chipmunks rely on large amounts of food hoarded during the preceding year; but there is no evidence of torpor expression. This kind of behavior is also observed in several hibernating ground squirrel species. Females that fail to reproduce may immerge up to several weeks before the others (Michener, 1978; Choromanski-Norris et al., 1986; Bintz, 1988; Neuhaus, 2000). An experiment in semi-natural conditions shows that female European ground squirrels (*Spermophilus citellus*) that were separated from males (and thus did not breed) entered into hibernation 4−6 weeks before females that were not separated (Millesi et al., 2008). These results support the view that hibernation phenology is influenced by a trade-off between reproduction and survival, where hibernation seems to provide benefits other than surviving periods of energetic stress. This trade-off may also explain differences (up to 1 month) in the timing of immergence and emergence between gender and age observed in rodents (Snyder et al., 1961; Holekamp and Nunes, 1989; Kawamichi, 1989; Sheriff et al., 2011; Kart Gür and Gür, 2015; Siutz et al., 2016) and bats (Stebbings, 1970; Thomson, 1982; Decher and Choate, 1995; Norquay and Willis, 2014).

So far, most studies have focused mainly on temperate hibernating species. However heterothermy that occurs during daily torpor and hibernation, is taxonomically and geographically widespread (Ruf and Geiser, 2015). A surprisingly large proportion of mammals, including a monotreme, several marsupials, and placental species regularly enter daily torpor and seasonal hibernation in the southern hemisphere (Grigg and Beard, 2000). For some species in these regions, the use of torpor may not be related to low environmental temperatures or limited food availability (Nowack et al., 2020). For example, the short-beaked echidna hibernates (Grigg et al., 1989) while ants and termites, which constitute the main part of its diet, remain available throughout the year (Grigg and Beard, 2000). Short-beaked echidnas are heavily armored, perhaps rendering avoidance of predation an unlikely adaptive benefit for hibernation. These observations suggest that some species may use hibernation because of the energy advantages provided by lack of activity, even though it is not an energy necessity for survival during a period of energetic stress (Grigg and Beard, 2000). Such case studies broaden the scope of possibilities for understanding the evolution of hibernation (Grigg and Beard, 2000; Ruf et al., 2012), and open up exciting perspectives for future research.

## Conclusion

Hibernation is considered an adaptation to seasonal, hence predictable decreases in food resources and ambient temperatures. However, hibernation is also observed in mild climates and when ambient conditions are still favorable for activity (Nowack et al., 2020). If remarkable physiological aspects of hibernation have been widely studied, fewer studies have focused on its ecological and evolutionary significance. Our study provides evidence that there may be a relationship between activity time budgets, hence the time dimension of allocation trade-off, and life history traits.

Our phylogenetic analyses show that variations in hibernation season duration can partially explain variations in longevity in hibernators. The models show a strong influence of phylogeny on this relationship and highlight the need for in-depth studies at an inter- and intra-population scales. For example, future studies may attempt to consider activity time budgets in the context of the pace of life syndrome by examining variations in hibernation with other physiological and behavioral traits. Our results, combined with information available in the literature, suggest that, in addition to its survival benefits during a period of energetic stress, hibernation season duration may have evolved to reduce the effects of other sources of extrinsic and intrinsic mortality.

## Author Contributions

TC conceived and drafted the manuscript and collected the data. SG and CH contributed to development of the concept and writing of the manuscript. TC, VV, and FD carried out the data analyses. VV, MT, PB, FD, CH, and SG substantially contributed to the conception. FD contributed to the English composition with many revisions of the manuscript. All authors contributed to the revisions.

## Conflict of Interest

The authors declare that the research was conducted in the absence of any commercial or financial relationships that could be construed as a potential conflict of interest.

## Footnotes

1 https://scholar.google.com

2 https://genomics.senescence.info/species/search_list.php

3 https://ecologicaldata.org/wiki/pantheria; Ecological Archives

4 https://academic.oup.com/mspecies

5 http://vertlife.org/phylosubsets/
