# Supplementary material for: Integrating Mortality Risk and the Adaptiveness of Hibernation
Source: Front Physiol. 2020 Jul 10;11:706. doi: 10.3389/fphys.2020.00706 (PMC7366871; doi:10.3389/fphys.2020.00706)
Supplement: Supplementary file 1 [file Image_1.pdf]

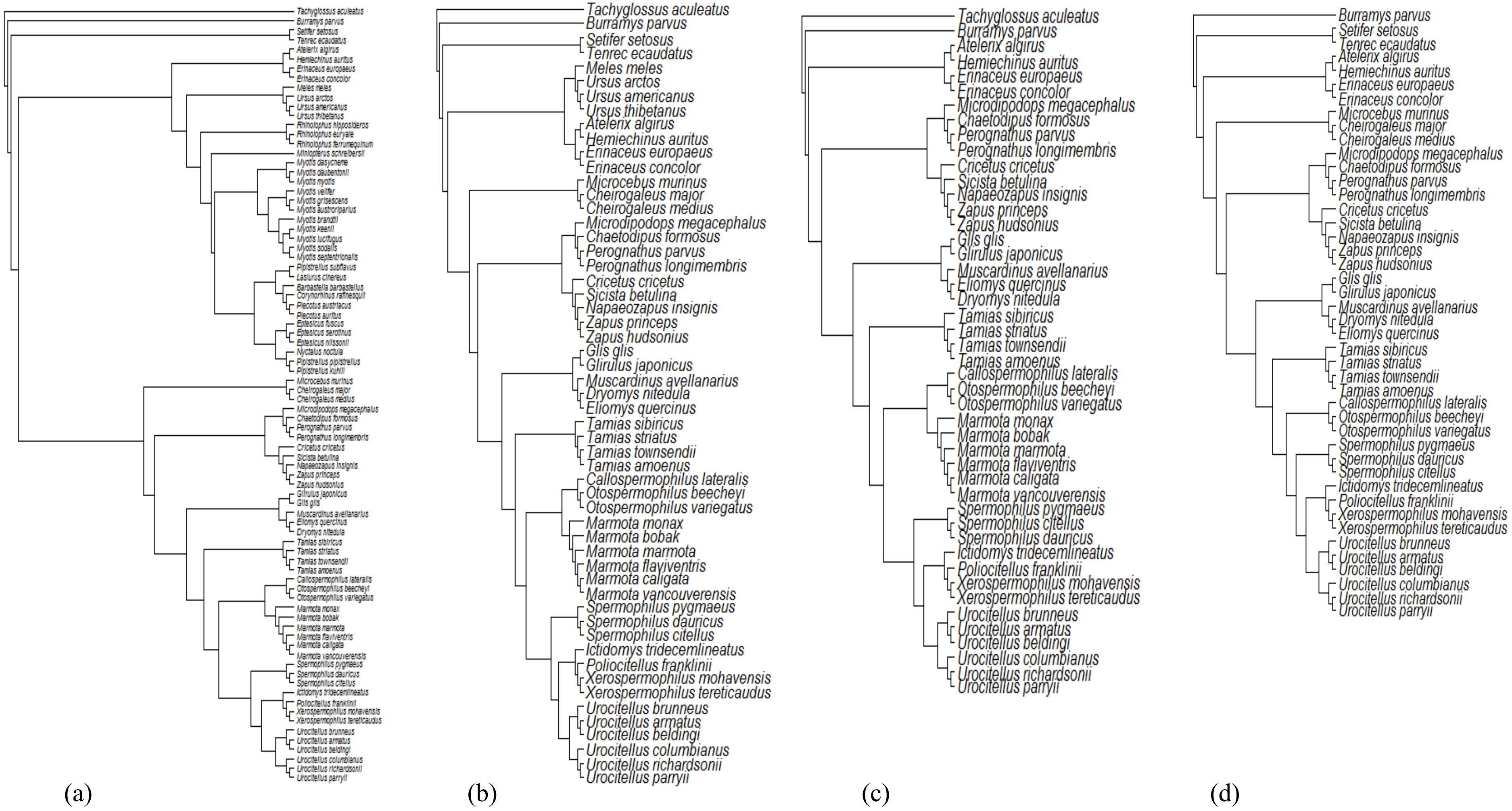

Figure S1. Consensus phylogenetic trees for the species under study: (a) all hibernators data set (N = 82), (b) the data set without bats (N =55), (c) the data set of deep hibernators without bats (N = 46) and (d) the data set of small hibernators (<1.5 kg) without bats (N = 44). Each consensus tree was built from 100 trees obtained from http://vertlife.org/phylosubsets/. Branch lengths were calculated using Grafen's computations with the 'ape' package in R (see Methods).
