# Supplementary material for: Integrating Mortality Risk and the Adaptiveness of Hibernation
Source: Front Physiol. 2020 Jul 10;11:706. doi: 10.3389/fphys.2020.00706 (PMC7366871; doi:10.3389/fphys.2020.00706)
Supplement: Supplementary file 2 [file Image_2.pdf]

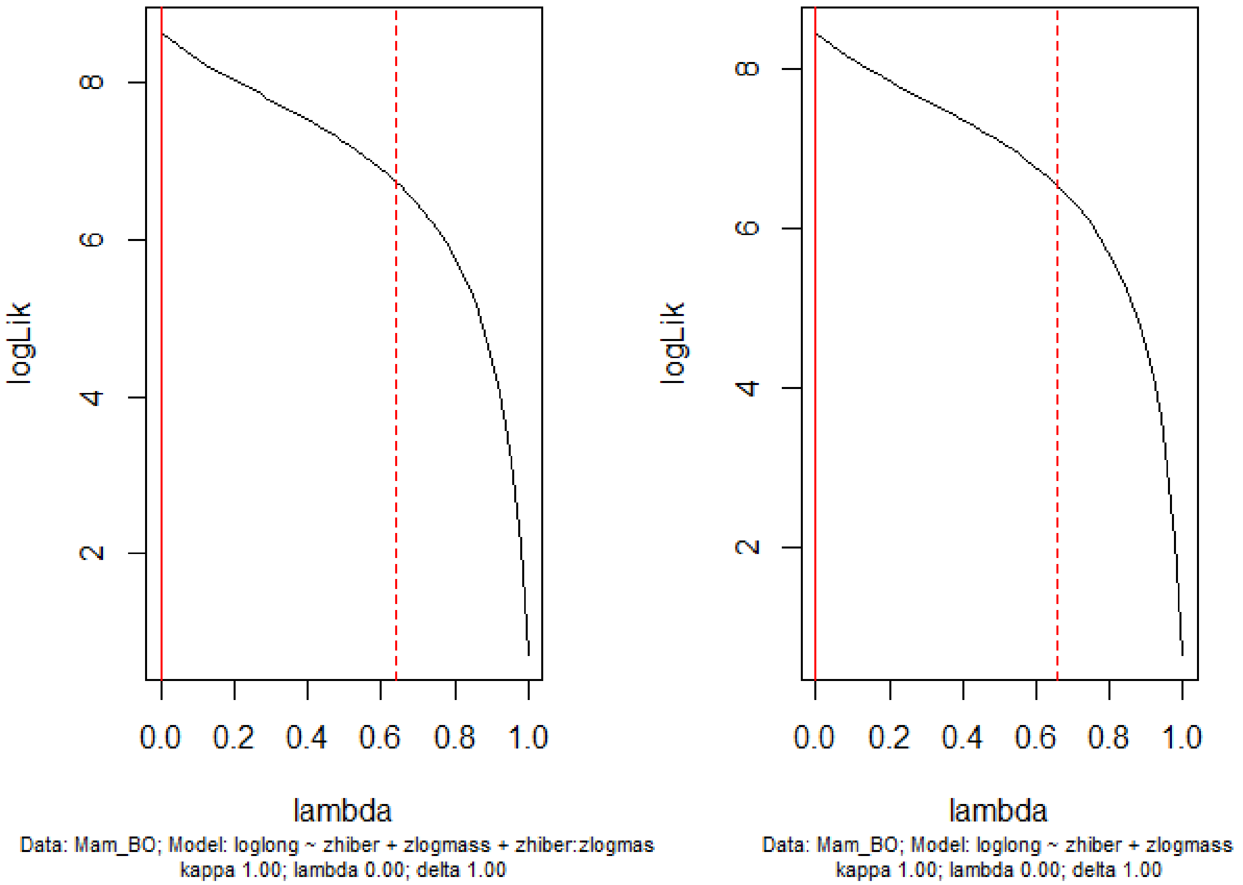

Figure S2: Likelihood profile of λs for bat models only (N = 27): (a) including the effects of hibernation season duration (standardized), body mass (standardized and log transformed) and interaction hibernation duration x body mass, (b) without interaction. logLik represent the likelihood value log-transformed. Lambda (λ) correspond to the relative effect of the phylogenetic tree on the linear model, ranging between 0 (covariation among species measurements is independent of co-ancestry) and 1 (covariance entirely explained by co-ancestry). Location of the maximum likelihood estimate of λ is symbolised by a red line, upper 95% CI is symbolized by a dashed red line. Note that the lower 95% CI can not be estimated.
