# Supplementary material for: Integrating Mortality Risk and the Adaptiveness of Hibernation
Source: Front Physiol. 2020 Jul 10;11:706. doi: 10.3389/fphys.2020.00706 (PMC7366871; doi:10.3389/fphys.2020.00706)
Supplement: Supplementary file 3 [file Table_1.docx]

| Species | Familly | Exclusion criteria | Longevity data | Hibernation data | Species included in study |
| --- | --- | --- | --- | --- | --- |
| Echinops telfairi | Afrosoricida | No | AnAge | No data | No |
| Ursus americanus | Carnivora | No | AnAge | Yes | Yes |
| Ursus arctos | Carnivora | No | AnAge | Yes | Yes |
| Ursus maritimus | Carnivora | Criteria 1 |  |  | No |
| Ursus thibetanus | Carnivora | No | AnAge | Yes | Yes |
| Antrozous pallidus | Chiroptera | No | AnAge | No data | No |
| Barbastella barbastellus | Chiroptera | No | AnAge | Yes | Yes |
| Corynorhinus rafinesquii | Chiroptera | No | PanTHERIA | Yes | Yes |
| Corynorhinus townsendii | Chiroptera | No | PanTHERIA | No data | No |
| Eptesicus fuscus | Chiroptera | No | AnAge | Yes | Yes |
| Eptesicus nilssonii | Chiroptera | No | AnAge | Yes | Yes |
| Eptesicus serotinus | Chiroptera | No | AnAge | Yes | Yes |
| Lasionycteris noctivagans | Chiroptera | No | AnAge | No data | No |
| Lasiurus cinereus | Chiroptera | No | AnAge | Yes | Yes |
| Miniopterus schreibersii | Chiroptera | No | AnAge | Yes | Yes |
| Myotis austroriparius | Chiroptera | No | PanTHERIA | Yes | Yes |
| Myotis bechsteinii | Chiroptera | No | AnAge | No data | No |
| Myotis blythii | Chiroptera | No | AnAge | No data | No |
| Myotis brandtii | Chiroptera | No | AnAge | Yes | Yes |
| Myotis californicus | Chiroptera | No | PanTHERIA | No data | No |
| Myotis dasycneme | Chiroptera | No | PanTHERIA | Yes | Yes |
| Myotis daubentonii | Chiroptera | No | AnAge | Yes | Yes |
| Myotis emarginatus | Chiroptera | No | AnAge | No data | No |
| Myotis evotis | Chiroptera | No | AnAge | No data | No |
| Myotis grisescens | Chiroptera | No | AnAge | Yes | Yes |
| Myotis keenii | Chiroptera | No | AnAge | Yes | Yes |
| Myotis leibii | Chiroptera | No | AnAge | No data | No |
| Myotis lucifugus | Chiroptera | No | AnAge | Yes | Yes |
| Myotis myotis | Chiroptera | No | AnAge | Yes | Yes |
| Myotis mystacinus | Chiroptera | No | AnAge | No data | No |
| Myotis nattereri | Chiroptera | No | AnAge | No data | No |
| Myotis sodalis | Chiroptera | No | AnAge | Yes | Yes |
| Myotis thysanodes | Chiroptera | No | AnAge | No data | No |
| Myotis velifer | Chiroptera | No | AnAge | Yes | Yes |
| Myotis volans | Chiroptera | No | AnAge | No data | No |
| Myotis yumanensis | Chiroptera | No | AnAge | No data | No |
| Nyctalus leisleri | Chiroptera | No | No data |  | No |
| Nyctalus noctula | Chiroptera | No | AnAge | Yes | Yes |
| Pipistrellus hesperus | Chiroptera | No | PanTHERIA | No data | No |
| Pipistrellus nathusii | Chiroptera | No | AnAge | No data | No |
| Pipistrellus pipistrellus | Chiroptera | No | AnAge | Yes | Yes |
| Pipistrellus subflavus | Chiroptera | No | AnAge | Yes | Yes |
| Plecotus auritus | Chiroptera | No | AnAge | Yes | Yes |
| Plecotus austriacus | Chiroptera | No | AnAge | Yes | Yes |
| Rhinolophus ferrumequinum | Chiroptera | No | AnAge | Yes | Yes |
| Rhinolophus hipposideros | Chiroptera | No | AnAge | Yes | Yes |
| Rhinolophus megaphyllus | Chiroptera | No | No data |  | No |
| Vespertilio murinus | Chiroptera | No | AnAge | No data | No |
| Zaedyus pichiy | Cingulata | No | AnAge | yes | No* |
| Acrobates pygmaeus | Diprotodontia | Criteria 3 |  |  | No |
| Burramys parvus | Diprotodontia | No | AnAge | Yes | Yes |
| Cercartetus nanus | Diprotodontia | Criteria 3 |  |  | No |
| Erinaceus concolor | Erinaceomorpha | No | AnAge | Yes | Yes |
| Erinaceus europaeus | Erinaceomorpha | No | AnAge | Yes | Yes |
| Hemiechinus auritus | Erinaceomorpha | No | AnAge | Yes | Yes |
| Paraechinus hypomelas | Erinaceomorpha | No | AnAge | No data | No |
| Dromiciops gliroides | Microbiotheria | No | No data |  | No |
| Tachyglossus aculeatus | Monotremata | No | AnAge | Yes | Yes |
| Cheirogaleus major | Primates | No | AnAge | Yes | Yes |
| Cheirogaleus medius | Primates | No | AnAge | Yes | Yes |
| Microcebus murinus | Primates | No | AnAge | Yes | Yes |
| Microcebus rufus | Primates | No | PanTHERIA | No data | No |
| Allactaga euphratica | Rodentia | No | AnAge | No data | No |
| Callospermophilus lateralis | Rodentia | No | AnAge | Yes | Yes |
| Callospermophilus saturatus | Rodentia | No | No data |  | No |
| Chaetodipus formosus | Rodentia | No | AnAge | Yes | Yes |
| Cricetus cricetus | Rodentia | No | AnAge | Yes | Yes |
| Cynomys gunnisoni | Rodentia | No | No data |  | No |
| Cynomys ludovicianus | Rodentia | Criteria 1 |  |  | No |
| Eliomys quercinus | Rodentia | No | AnAge | Yes | Yes |
| Glis glis | Rodentia | No | Other source | Yes | Yes |
| Graphiurus murinus | Rodentia | No | AnAge | No data | No |
| Jaculus jaculus | Rodentia | No | AnAge | No data | No |
| Marmota bobak | Rodentia | No | PanTHERIA | Yes | Yes |
| Marmota flaviventris | Rodentia | No | AnAge | Yes | Yes |
| Marmota marmota | Rodentia | No | AnAge | Yes | Yes |
| Marmota monax | Rodentia | No | AnAge | Yes | Yes |
| Marmota vancouverensis | Rodentia | No | AnAge | Yes | Yes |
| Mesocricetus auratus | Rodentia | No | AnAge | No data | No |
| Microdipodops megacephalus | Rodentia | No | Other source | Yes | Yes |
| Muscardinus avellanarius | Rodentia | No | Other source | Yes | Yes |
| Napaeozapus insignis | Rodentia | No | PanTHERIA | Yes | Yes |
| Otospermophilus beecheyi | Rodentia | No | Other source | Yes | Yes |
| Perognathus flavescens | Rodentia | No | No data |  | No |
| Perognathus flavus | Rodentia | No | PanTHERIA | No data | No |
| Perognathus longimembris | Rodentia | No | AnAge | Yes | Yes |
| Perognathus parvus | Rodentia | No | AnAge | Yes | Yes |
| Sicista betulina | Rodentia | No | Other source | Yes | Yes |
| Spermophilus citellus | Rodentia | No | AnAge | Yes | Yes |
| Spermophilus dauricus | Rodentia | No | Other source | Yes | Yes |
| Tamias amoenus | Rodentia | No | Other source | Yes | Yes |
| Tamias minimus | Rodentia | No | AnAge | No data | No |
| Tamias sibiricus | Rodentia | No | AnAge | Yes | Yes |
| Tamias striatus | Rodentia | No | AnAge | Yes | Yes |
| Tamias townsendii | Rodentia | No | AnAge | Yes | Yes |
| Urocitellus armatus | Rodentia | No | Other source | Yes | Yes |
| Urocitellus beldingi | Rodentia | No | Other source | Yes | Yes |
| Urocitellus columbianus | Rodentia | No | Other source | Yes | Yes |
| Urocitellus parryii | Rodentia | No | Other source | Yes | Yes |
| Urocitellus richardsonii | Rodentia | No | Other source | Yes | Yes |
| Urocitellus townsendii | Rodentia | No | No data |  | No |
| Zapus hudsonius | Rodentia | No | AnAge | Yes | Yes |
| Zapus princeps | Rodentia | No | Other source | Yes | Yes |
| Amblysomus hottentotus | Afrosoricida | No | No data |  | No |
| Setifer setosus | Afrosoricida | No | AnAge | Yes | Yes |
| Tenrec ecaudatus | Afrosoricida | No | AnAge | Yes | Yes |
| Meles meles | Carnivora | No | AnAge | Yes | Yes |
| Chalinolobus gouldii | Chiroptera | No | No data |  | No |
| Hipposideros terasensis | Chiroptera | No | No data |  | No |
| Lasiurus borealis | Chiroptera | No | No data |  | No |
| Mops condylurus | Chiroptera | No | No data |  | No |
| Myotis adversus | Chiroptera | No | No data |  | No |
| Nyctophilus bifax | Chiroptera | No | No data |  | No |
| Nyctophilus geoffroyi | Chiroptera | No | No data |  | No |
| Nyctophilus gouldi | Chiroptera | No | No data |  | No |
| Rhinopoma microphyllum | Chiroptera | No | No data |  | No |
| Tadarida aegyptiaca | Chiroptera | No | No data |  | No |
| Tadarida brasiliensis | Chiroptera | No | AnAge | No data | No |
| Tadarida teniotis | Chiroptera | No | AnAge | No data | No |
| Vespadelus vulturnus | Chiroptera | No | No data |  | No |
| Cercartetus concinnus | Diprotodontia | Criteria 3 |  |  | No |
| Cercartetus lepidus | Diprotodontia | Criteria 3 |  |  | No |
| Atelerix algirus | Eulipotyphla | No | PanTHERIA | Yes | Yes |
| Atelerix frontalis | Eulipotyphla | No | No data |  | No |
| Elephantulus edwardii | Macroscelidea | Criteria 3 |  |  | No |
| Elephantulus myurus | Macroscelidea | Criteria 3 |  |  | No |
| Cheirogaleus crossleyi | Primates | No | No data |  | No |
| Microcebus griseorufus | Primates | No | No data |  | No |
| Allactaga williamsi | Rodentia | No | No data |  | No |
| Cynomys parvidens | Rodentia | No | No data |  | No |
| Cynomys leucurus | Rodentia | No | No data |  | No |
| Glirulus japonicus | Rodentia | No | AnAge | Yes | Yes |
| Graphiurus ocularis | Rodentia | No | No data |  | No |
| Ictidomys tridecemlineatus | Rodentia | No | AnAge | Yes | Yes |
| Ictidomys mexicanus | Rodentia | No | No data |  | No |
| Jaculus orientalis | Rodentia | No | AnAge | No data | No |
| Marmota broweri | Rodentia | No | No data |  | No |
| Mesocricetus brandti | Rodentia | No | AnAge | No data | No |
| Microdipodops pallidus | Rodentia | No | No data |  | No |
| Otospermophilus variegatus | Rodentia | No | AnAge | Yes | Yes |
| Spermophilus xanthoprymnus | Rodentia | No | No data |  | No |
| Urocitellus elegans | Rodentia | No | No data |  | No |
| Xerospermophilus tereticaudus | Rodentia | No | AnAge | Yes | Yes |
| Myotis septentrionalis | Chiroptera | No | Other source | Yes | Yes |
| Myotis kuhlii | Chiroptera | No | AnAge | Yes | Yes |
| Rhinolophus euryale | Chiroptera | No | Other source | Yes | Yes |
| Dryomys nitedula | Rodentia | No | Other source | Yes | Yes |
| Marmota caligata | Rodentia | No | AnAge | Yes | Yes |
| Poliocitellus franklinii | Rodentia | No | AnAge | Yes | Yes |
| Spermophilus pygmaeus | Rodentia | No | AnAge | Yes | Yes |
| Urocitellus brunneus | Rodentia | No | Other source | Yes | Yes |
| Xerospermophilus mohavensis | Rodentia | No | AnAge | Yes | Yes |

Table S1: Summary of the hibernators selected in this study among the 152 listed hibernators. Species (1) in black are listed in Turbill et al (2011), (2) in dark blue represent the other species listed by Ruf and Geiser (2015) and (3) in light blue represent other known hibernators. AnAge refers to AnAge data base (The Animal Aging and Longevity Database; https://genomics.senescence.info/species/search_list.php; Human Ageing and Genomic Resources; Magalhães and Costa, 2009), and PanTHERIA refers to the PanTHERIA data base (https://ecologicaldata.org/wiki/PanTHERIA; Ecological Archives, Ecological Society of America; Jones et al., 2009). We mention the new nomenclature for ground squirrel species, which may not correspond to the nomenclature of the lists presented. Some species were first excluded according to the exclusion criteria listed in the publication. We then investigate the longevity of hibernators according to the methods described in the publication. Only species for which we have found both the hibernation season duration and longevity were included in the study. All references for longevity and hibernation duration data are presented in Table S2.

*Note that longevity (12.5 years) and hibernation season duration (90 day) of *Zaedyus pichiy* are known (Magalhaes and Costa, 2009; Superina and Abba, 2014) but by including this species the binary condition of the phylogenetic tree was not satisfied. A rooted tree is considered binary if all nodes (including the root node) have exactly two descendant nodes. This condition was tested using the ‘ape 5.0’ packages in R v. 3.6.2.

De Magalhaes, J. P., & Costa, J. (2009). A database of vertebrate longevity records and their relation to other life‐history traits. Journal of evolutionary biology, 22(8), 1770-1774.

Jones, K. E., Bielby, J., Cardillo, M., Fritz, S. A., O'Dell, J., Orme, C. D. L., ... & Connolly, C. (2009). PanTHERIA: a species‐level database of life history, ecology, and geography of extant and recently extinct mammals: Ecological Archives E090‐184. Ecology, 90(9), 2648-2648.

Ruf, T., & Geiser, F. (2015). Daily torpor and hibernation in birds and mammals. Biological Reviews, 90(3), 891-926.

Superina, M., & Abba, A. M. (2014). *Zaedyus pichiy* (Cingulata: Dasypodidae). Mammalian Species, 46(905), 1-10.

Turbill, C., Bieber, C., & Ruf, T. (2011). Hibernation is associated with increased survival and the evolution of slow life histories among mammals. *Proceedings of the Royal Society B: Biological Sciences*, *278*(1723), 3355-3363.
