# Supplementary material for: Integrating Mortality Risk and the Adaptiveness of Hibernation
Source: Front Physiol. 2020 Jul 10;11:706. doi: 10.3389/fphys.2020.00706 (PMC7366871; doi:10.3389/fphys.2020.00706)
Supplement: Supplementary file 4 [file Table_2.docx]

| **Species** | **Family** | **Hibernation season duration (day)** | **Body mass (g)** | | **Longevity (year)** | **Arboreality** | **Wild or Captivity** |
| --- | --- | --- | --- | --- | --- | --- | --- |
| Burramys parvus | Burramyidae | 210 ^1,2^ | | 45 ^3^ | 12 ^3^ | Yes ^4^ | Wild |
| Cheirogaleus major | Cheirogaleidae | 150 ^5–7^ | | 395 ^3^ | 13.4 ^3^ | Yes ^8,9^ | Captivity |
| Cheirogaleus medius | Cheirogaleidae | 210 ^10^ | | 380 ^3^ | 29 ^3^ | Yes ^4,8,11^ | Captivity |
| Microcebus murinus | Cheirogaleidae | 186 ^12^ | | 64.8 ^3^ | 18.2 ^3^ | Yes ^4,13^ | Captivity |
| Cricetus cricetus | Cricetidae | 180 ^14^ | | 506.7 ^3^ | 3.6 ^3^ | No ^15^ | Captivity |
| Napaeozapus insignis | Dipodidae | 210 ^16^ * | | 22.25 ^17^ | 4 ^17^ | No ^18^ | Wild |
| Sicista betulina | Dipodidae | 210 ^19^ * | | 8.92 ^17^ | 3.5 ^20^ | No ^9^ | Wild |
| Zapus hudsonius | Dipodidae | 265 ^21^ | | 18 ^3^ | 5.6 ^3^ | No ^22^ | Captivity |
| Zapus princeps | Dipodidae | 296 ^23^ | | 27.2 ^17^ | 6 ^24^ | No ^25^ | Wild |
| Atelerix algirus | Erinaceidae | 120 ^26^ | | 958 ^17^ | 7 ^17^ | No ^27^ | Captivity |
| Erinaceus concolor | Erinaceidae | 210 ^28^ | | 719 ^3^ | 7 ^3^ | No ^9^ | Captivity |
| Erinaceus europaeus | Erinaceidae | 225 ^29^ | | 750 ^3^ | 11.7 ^3^ | No ^4^ | Captivity |
| Hemiechinus auritus | Erinaceidae | 150 ^30^ | | 342 ^3^ | 7.6 ^3^ | No ^9^ | Captivity |
| Dryomys nitedula | Gliridae | 240 ^31^ | | 26 ^3^ | 8 ^31^ | Yes ^9,32^ | Wild |
| Eliomys quercinus | Gliridae | 210 ^33^ | | 82.5 ^3^ | 5.5 ^3^ | Yes ^9,34^ | Captivity |
| Glirulus japonicus | Gliridae | 195 ^35^ | | 27 ^3^ | 7 ^3^ | Yes ^9,36^ | Captivity |
| Glis glis | Gliridae | 282 ^37^ | | 125 ^3^ | 13 ^38^ | Yes ^9,39^ | Wild |
| Muscardinus avellanarius | Gliridae | 210 ^40^ | | 27.3 ^3^ | 6 ^40,41^ | Yes ^9,42^ | Wild |
| Chaetodipus formosus | Heteromyidae | 105 ^43^ | | 19 ^3^ | 7.1 ^3^ | No ^9^ | Captivity |
| Microdipodops megacephalus | Heteromyidae | 150 ^44^ | | 12.3 ^17^ | 5.42 ^17^ | No ^45^ | Captivity |
| Perognathus longimembris | Heteromyidae | 195 ^43^ | | 8 ^3^ | 8.3 ^3^ | No ^9^ | Captivity |
| Perognathus parvus | Heteromyidae | 120 ^46^ | | 20.1 ^3^ | 5.8 ^3^ | No ^47^ | Captivity |
| Miniopterus schreibersii | Miniopteridae | 150 ^48^ * | | 13 ^3^ | 22 ^3^ | No ^4^ | Wild |
| Meles meles | Mustelidae | 180 ^49^ * | | 13000 ^3^ | 18.6 ^3^ | No ^4^ | Captivity |
| Rhinolophus euryale | Rhinolophidae | 150 ^50–52^ | | 8.2 ^3^ | 21 ^53^ | No ^9^ | Wild |
| Rhinolophus ferrumequinum | Rhinolophidae | 225 ^54^ | | 22.875 ^3^ | 30.5 ^3^ | No ^4^ | Wild |
| Rhinolophus hipposideros | Rhinolophidae | 210 ^55^ | | 4.6 ^3^ | 29.4 ^3^ | No ^4^ | Wild |
| Callospermophilus lateralis | Sciuridae | 274 ^56^ | | 157.6 ^3^ | 10.4 ^3^ | No ^57^ | Captivity |
| Ictidomys tridecemlineatus | Sciuridae | 225 ^58^ | | 172.7 ^3^ | 7.9 ^3^ | No ^59^ | Captivity |
| Marmota bobak | Sciuridae | 240 ^60^ * | | 7300 ^17^ | 15 ^17^ | No ^9^ | Wild |
| Marmota caligata | Sciuridae | 240 ^61^ * | | 4300 ^3^ | 12.1 ^3^ | No ^61^ | Captivity |
| Marmota flaviventris | Sciuridae | 240 ^62^ | | 3500 ^3^ | 21.2 ^3^ | No ^63^ | Captivity |
| Marmota marmota | Sciuridae | 195 ^64^ | | 3500 ^3^ | 17.4 ^3^ | No ^4^ | Captivity |
| Marmota monax | Sciuridae | 166.9 ^65^ | | 4000 ^3^ | 14 ^3^ | No ^4^ | Captivity |
| Marmota vancouverensis | Sciuridae | 210 ^66^ | | 4750 ^3^ | 12.1 ^3^ | No ^4^ | Captivity |
| Otospermophilus beecheyi | Sciuridae | 210 ^67^ | | 508.5 ^68^ | 5 ^53^ | No ^69^ | Wild |
| Otospermophilus variegatus | Sciuridae | 210 ^70^ * | | 663 ^3^ | 9.8 ^3^ | Yes ^71–74^ | Captivity |
| Poliocitellus franklinii | Sciuridae | 255 ^75^ | | 459 ^3^ | 7.2 ^3^ | No ^76^ | Captivity |
| Spermophilus citellus | Sciuridae | 225 ^77^ | | 217 ^3^ | 6.7 ^3^ | No ^4^ | Captivity |
| Spermophius dauricus | Sciuridae | 210 ^78^ * | | 223.8 ^68^ | 7 ^78^ | No ^9^ | Wild |
| Spermophilus pygmaeus | Sciuridae | 240 ^79^ | | 136 ^3^ | 7.1 ^3^ | No ^9^ | Captivity |
| Tamias amoenus | Sciuridae | 150 ^80^ | | 50.63 ^17^ | 5.17 ^81^ | Yes ^9,82–84^ | Wild |
| Tamias sibiricus | Sciuridae | 210 ^85^ | | 85 ^3^ | 9.6 ^3^ | Yes ^9,84–86^ | Captivity |
| Tamias striatus | Sciuridae | 210 ^87^ | | 96 ^3^ | 9.5 ^3^ | Yes ^9,84,88–90^ | Captivity |
| Tamias townsendii | Sciuridae | 135 ^91^ | | 75 ^3^ | 9.3 ^3^ | Yes ^9,84,92–94^ | Captivity |
| Urocitellus armatus | Sciuridae | 289 ^95^ | | 306.48 ^17^ | 7 ^96^ | No ^97^ | Wild |
| Urocitellus beldingi | Sciuridae | 270 ^98^ | | 265.2 ^68^ | 12 ^99^ | No ^100^ | Wild |
| Urocitellus brunneus | Sciuridae | 240 ^101^ | | 300 ^17^ | 8 ^101^ | No ^102^ | Wild |
| Urocitellus columbianus | Sciuridae | 285 ^103^ | | 470.94^17^ | 11 ^104^ | No ^105^ | Wild |
| Urocitellus parryii | Sciuridae | 240 ^106^ | | 524.3 ^68^ | 10 ^107^ | No ^108^ | Wild |
| Urocitellus richardsonii | Sciuridae | 258 ^109^ | | 325.1 ^17^ | 6 ^110^ | No ^111^ | Wild |
| Xerospermophilus mohavensis | Sciuridae | 210 ^112^ * | | 150 ^3^ | 7.8 ^3^ | No ^113^ | Captivity |
| Xerospermophilus tereticaudus | Sciuridae | 240 ^114^ | | 163.3 ^3^ | 8.9 ^3^ | No ^112^ | Captivity |
| Tachyglossus aculeatus | Tachyglossidae | 180 ^115^ | | 3500 ^3^ | 49.5 ^3^ | No ^4^ | Captivity |
| Setifer setosus | Tenrecidae | 210 ^116^ | | 225 ^3^ | 14.1 ^3^ | Yes ^4,117,118^ | Captivity |
| Tenrec ecaudatus | Tenrecidae | 270 ^119^ | | 900 ^3^ | 8.7 ^3^ | No ^120^ | Captivity |
| Ursus americanus | Ursidae | 210 ^121^ * | | 154250 ^3^ | 34 ^3^ | No ^4^ | Captivity |
| Ursus arctos | Ursidae | 215 ^122^ | | 277500 ^3^ | 40 ^3^ | No ^4^ | Captivity |
| Ursus thibetanus | Ursidae | 150 ^123^ | | 103750 ^3^ | 39.2 ^3^ | Yes ^4,124,125^ | Captivity |
| Barbastella barbastellus | Vespertilionidae | 225 ^126^ | | 10.25 ^3^ | 23 ^3^ | No ^4^ | Wild |
| Corynorhinus rafinesquii | Vespertilionidae | 135 ^127^ | | 9.15 ^17^ | 10.1 ^17^ | No ^128^ | Wild |
| Eptesicus fuscus | Vespertilionidae | 180 ^129^ | | 23 ^3^ | 19 ^3^ | No ^4^ | Wild |
| Eptesicus nilssonii | Vespertilionidae | 180 ^130^ * | | 13 ^3^ | 20 ^3^ | No ^4^ | Wild |
| Eptesicus serotinus | Vespertilionidae | 210 ^131^ | | 18.2 ^3^ | 21 ^3^ | No ^4^ | Wild |
| Lasiurus cinereus | Vespertilionidae | 180 ^132^ | | 24 ^3^ | 14 ^3^ | No ^4^ | Wild |
| Myotis austroriparius | Vespertilionidae | 210 ^133,134^ * | | 7.35 ^17^ | 6 ^17^ | No ^133^ | Wild |
| Myotis brandtii | Vespertilionidae | 255 ^135^ * | | 7 ^3^ | 41 ^3^ | No ^4^ | Wild |
| Myotis dasycneme | Vespertilionidae | 195 ^136^ * | | 15.16 ^17^ | 19.5 ^17^ | No ^4^ | Wild |
| Myotis daubentonii | Vespertilionidae | 240 ^137^ | | 8.5 ^3^ | 28 ^3^ | No ^4^ | Wild |
| Myotis grisescens | Vespertilionidae | 120 ^138^ * | | 9.25 ^3^ | 16.5 ^3^ | No ^4^ | Wild |
| Myotis keenii | Vespertilionidae | 180 ^139^ | | 7.4 ^3^ | 19 ^3^ | No ^4^ | Wild |
| Myotis lucifugus | Vespertilionidae | 241 ^140^ | | 10 ^3^ | 34 ^3^ | No ^4^ | Wild |
| Myotis myotis | Vespertilionidae | 150 ^141^ | | 28.55 ^3^ | 37.1 ^3^ | No ^4^ | Wild |
| Myotis septentrionalis | Vespertilionidae | 180 ^142^ | | 7 ^143^ | 19 ^144^ | No ^145^ | Wild |
| Myotis sodalis | Vespertilionidae | 210 ^146^ | | 7.7 ^3^ | 20 ^3^ | No ^4^ | Wild |
| Myotis velifer | Vespertilionidae | 180 ^147^ | | 10.1 ^3^ | 11.3 ^3^ | No ^4^ | Wild |
| Nyctalus noctula | Vespertilionidae | 150 ^52,148^ | | 27.75 ^3^ | 12 ^3^ | No ^4^ | Wild |
| Pipistrellus kuhlii | Vespertilionidae | 150 ^149^* | | 6 ^3^ | 8 ^3^ | No ^4^ | Wild |
| Pipistrellus pipistrellus | Vespertilionidae | 150 ^52,150^ | | 5 ^3^ | 16.6 ^3^ | No ^4^ | Wild |
| Pipistrellus subflavus | Vespertilionidae | 180 ^151^ | | 7.5 ^3^ | 14.8 ^3^ | No ^4^ | Wild |
| Plecotus auritus | Vespertilionidae | 150 ^152^ | | 7.8 ^3^ | 30 ^3^ | No ^4^ | Wild |
| Plecotus austriacus | Vespertilionidae | 180 ^152^ | | 12 ^3^ | 25.5 ^3^ | No ^4^ | Wild |

Table S2: Data on hibernation season duration, body mass, longevity and arboreality lifestyle of all mammals studied in the different models. Hibernation season duration with (*) corresponds to publications for which the methodology used to determine this duration could not be verified. The “Wild or Captivity” parameter refers to the source of longevity data. The “arboreality” parameter distinguishes between species considered arboreal and semi-arboreal (Yes) and non-arboreal (No). Brown bear (*Ursus arctos*) and American black bear (*Ursus americanus*) have been defined as non-arboreal, as in Healy et al, 2014, as the majority of adults are losing this lifestyle ^153^ contrary to Asian black bear (*Ursus thibetanus*) ^124^. Bats were considered to be non-arboreal even though it nests in trees as nesting in a protected area is a common characteristic of all bats and therefore already taken into account in the “bat (yes/no)” factor.

1. Körtner, G. & Geiser, F. Ecology of natural hibernation in the marsupial mountain pygmy-possum (Burramys parvus). *Oecologia* **113**, 170–178 (1998).

2. Körtner, G., Song, X. & Geiser, F. Rhythmicity of torpor in a marsupial hibernator, the mountain pygmy-possum (Burramysparvus), under natural and laboratory conditions. *Journal of comparative Physiology B* **168**, 631–638 (1998).

3. Magalhães, J. P. D. & Costa, J. A database of vertebrate longevity records and their relation to other life-history traits. *Journal of Evolutionary Biology* **22**, 1770–1774 (2009).

4. Healy, K. *et al.* Ecology and mode-of-life explain lifespan variation in birds and mammals. *Proceedings of the Royal Society B: Biological Sciences* **281**, 20140298 (2014).

5. Wright, P. C. & Martin, L. B. Predation, Pollination and Torpor in Two Nocturnal Prosimians: Cheirogaleus Major and Microcebus Rufus in the Rain Forest of Madagascar. in *Creatures of the Dark: The Nocturnal Prosimians* (eds. Alterman, L., Doyle, G. A. & Izard, M. K.) 45–60 (Springer US, 1995). doi:10.1007/978-1-4757-2405-9_4.

6. Lahann, P. Biology of Cheirogaleus major in a Littoral Rain Forest in Southeast Madagascar. *Int J Primatol* **28**, 895–905 (2007).

7. Schülke, O. & Ostner, J. Physiological ecology of cheirogaleid primates: variation in hibernation and torpor. *Acta Ethologica* **10**, 13–21 (2007).

8. Clutton‐Brock, T. H. & Harvey, P. H. Primates, brains and ecology. *Journal of Zoology* **190**, 309–323 (1980).

9. Hidasi‐Neto, J., Loyola, R. & Cianciaruso, M. V. Global and local evolutionary and ecological distinctiveness of terrestrial mammals: identifying priorities across scales. *Diversity and Distributions* **21**, 548–559 (2015).

10. Dausmann, K. H., Glos, J., Ganzhorn, J. U. & Heldmaier, G. Hibernation in a tropical primate. *Nature* **429**, 825–826 (2004).

11. Lemelin, P. & Schmitt, D. Seasonal variation in body mass and locomotor kinetics of the fat-tailed dwarf lemur (Cheirogaleus medius). *Journal of Morphology* **260**, 65–71 (2004).

12. Schmid, J. & Kappeler, P. M. Fluctuating sexual dimorphism and differential hibernation by sex in a primate, the gray mouse lemur (Microcebus murinus). *Behavioral Ecology and Sociobiology* **43**, 125–132 (1998).

13. Toussaint, S. *et al.* Food acquisition on arboreal substrates by the grey mouse lemur: implication for primate grasping evolution. *Journal of Zoology* **291**, 235–242 (2013).

14. Siutz, C., Franceschini, C. & Millesi, E. Sex and age differences in hibernation patterns of common hamsters: adult females hibernate for shorter periods than males. *Journal of Comparative Physiology B* **186**, 801–811 (2016).

15. Kryštufek, B., Hoffmann, I. E., Nedyalkov, N., Pozdnyakov, A. & Vohralík, V. Cricetus cricetus (Rodentia: Cricetidae). *Mamm Species* **52**, 10–26 (2020).

16. Wrigley, R. E. *Systematics and biology of the woodland jumping mouse, Napaeozapus insignis 47*. (Urbana, University of Illinois Press, 1972).

17. Jones, K. E. *et al.* PanTHERIA: a species-level database of life history, ecology, and geography of extant and recently extinct mammals. *Ecology* **90**, 2648–2648 (2009).

18. Whitaker, J. O. & Wrigley, R. E. Napaeozapus insignis. *Mamm Species* 1–6 (1972) doi:10.2307/3503916.

19. Johansen, K. & Krog, J. Diurnal body temperature variations and hibernation in the birchmouse, Sicista betulina. *American Journal of Physiology-Legacy Content* **196**, 1200–1204 (1959).

20. Carey, J. R. & Judge, D. S. *Life Spans of Mammals, Birds, Amphibians, Reptiles, and Fish*. vol. 8 (2000).

21. Hoyle, J. & Boonstra, R. Life history traits of the meadow jumping mouse, Zapus hudsonius, in Southern Ontario. (1986).

22. Whitaker, J. O. Zapus hudsonius. *Mamm Species* 1–7 (1972) doi:10.2307/3504066.

23. Cranford, J. A. Hibernation in the Western Jumping Mouse (Zapus princeps). *J Mammal* **59**, 496–509 (1978).

24. Falk, J. W. & Millar, J. S. Reproduction by female Zapus princeps in relation to age, size, and body fat. *Can. J. Zool.* **65**, 568–571 (1987).

25. Hart, E. B., Belk, M. C., Jordan, E. & Gonzalez, M. W. Zapus princeps. *Mamm Species* 1–7 (2004) doi:10.1644/749.

26. Mouhoub sayah, C. *et al.* Road mortality of the Algerian hedgehog (Atelerix algirus) in the Soummam Valley (Algeria). *Revue d’écologie* (2009).

27. Nowak, R. M. & Walker, E. P. *Walker’s Mammals of the World*. (JHU Press, 1999).

28. Ahmet, Ö. Some biological, ecological and behavioural features of Erinaceus concolor Martin, 1838 (Mammalia: Insectivora) in Turkey. *Gazi University Journal of Science* **19**, 91–97 (2006).

29. Rautio, A., Valtonen, A., Auttila, M. & Kunnasranta, M. Nesting patterns of European hedgehogs (Erinaceus europaeus) under northern conditions. *Acta theriologica* **59**, 173–181 (2014).

30. SCHOENFELD, M. & Yoram, Y.-T. The biology of two species of hedgehogs, Erinaceus europaeus concolor and Hemiechinus auritus aegyptius, in Israel. *Mammalia* **49**, 339–356 (1985).

31. Juškaitis, R. Ecology of the forest dormouse Dryomys nitedula (Pallas 1778) on the north-western edge of its distributional range. *Mammalia* **79**, 33–41 (2015).

32. Juškaitis, R., Balčiauskas, L. & Šiožinytė, V. Nest site preference of forest dormouse Dryomys nitedula (Pallas) in the north-western corner of the distribution range. *Pol. J. Ecol* **60**, 815–826 (2012).

33. Bertolino, S., Viano, C. & Currado, I. Population dynamics, breeding patterns and spatial use of the garden dormouse (Eliomys quercinus) in an Alpine habitat. *Journal of Zoology* **253**, 513–521 (2001).

34. Bertolino, S. & Montezemolo, N. C. di. Garden dormouse (Eliomys quercinus) nest site selection in an alpine habitat. *Ethology Ecology & Evolution* **19**, 51–60 (2007).

35. Nakamura-Kojo, Y., Kojo, N., Ootsuka, T., Minami, M. & Tamate, H. B. Influence of tree resources on nest box use by the Japanese dormouse Glirulus japonicus. *Mammal study* **39**, 17–26 (2014).

36. Minato, S. & Doei, H. Arboreal activity of Glirulus japonicus (Rodentia: Myoxidae) confirmed by use of bryophytes as nest materials. *Acta Theriologica* **40**, 309–314 (1995).

37. Hoelzl, F. *et al.* How to spend the summer? Free-living dormice (Glis glis) can hibernate for 11 months in non-reproductive years. *J Comp Physiol B* **185**, 931–939 (2015).

38. Trout, R. C., Brooks, S. & Morris, P. Nest box usage by old edible dormice (Glis glis) in breeding and non-breeding years. *fozo* **64**, 320–324 (2015).

39. Kryštufek, B. Glis glis (Rodentia: Gliridae). *Mamm Species* **42**, 195–206 (2010).

40. Juškaitis, R. Life tables for the common dormouse Muscardinus avellanarius in Lithuania. *Acta Theriologica* **44**, 465–470 (1999).

41. Juškaitis, R. Summer mortality in the hazel dormouse (Muscardinus avellanarius) and its effect on population dynamics. *Acta Theriol* **59**, 311–316 (2014).

42. Bright, P. W. Behaviour of specialist species in habitat corridors: arboreal dormice avoid corridor gaps. *Animal Behaviour* **56**, 1485–1490 (1998).

43. Kenagy, G. J. & Bartholomew, G. A. Seasonal Reproductive Patterns in Five Coexisting California Desert Rodent Species: Ecological Archives M055-002. *Ecological Monographs* **55**, 371–397 (1985).

44. O’Farrell, M. J. Seasonal activity patterns of rodents in a sagebrush community. *Journal of Mammalogy* **55**, 809–823 (1974).

45. O’Farrell, M. J. & Blaustein, A. R. Microdipodops megacephalus. *Mamm Species* **46**, 1–3 (1974).

46. O’Farrell, T. P., Olson, R. J., Gilbert, R. O. & Hedlund, J. D. A Population of Great Basin Pocket Mice, Perognathus parvus, in the Shrub-Steppe of South-Central Washington. *Ecological Monographs* **45**, 1–28 (1975).

47. Verts, B. J. & Kirkland, G. L. Perognathus parvus. *Mamm Species* 1–8 (1988) doi:10.2307/3504324.

48. Oxberry, B. A. Female reproductive patterns in hibernating bats. *Reproduction* **56**, 359–367 (1979).

49. Kowalczyk, R., Jȩdrzejewska, B. & Zalewski, A. Annual and circadian activity patterns of badgers (Meles meles) in Białowieża Primeval Forest (eastern Poland) compared with other Palaearctic populations. *Journal of Biogeography* **30**, 463–472 (2003).

50. Malinčiová, L., Hrehová, L., Maxinová, E., Uhrin, M. & Pristaš, P. The dynamics of Mediterranean horseshoe bat (Rhinolophus euryale, Chiroptera) gut microflora during hibernation. https://www.ingentaconnect.com/content/miiz/actac/2017/00000019/00000001/art00017 (2017) doi:info:doi/10.3161/15081109ACC2017.19.1.017.

51. Miková, E., Varcholová, K., Boldogh, S. & Uhrin, M. Winter diet analysis in Rhinolophus euryale (Chiroptera). *Open Life Sciences* **8**, 848–853 (2013).

52. Arthur, L. & Lemaire, M. *Les Chauves-souris de France Belgique Luxembourg et Suisse*. (Biotope, 2009).

53. Fitch, H. S. Ecology of the California Ground Squirrel on Grazing Lands. *The American Midland Naturalist* **39**, 513–596 (1948).

54. Ransome, R. D. The distribution of the Greater horse-shoe bat, Rhinolophus ferrum-equinum, during hibernation, in relation to environmental factors. *Journal of Zoology* **154**, 77–112 (1968).

55. Harmata, W. The length of awakening time from hibernation of three species of bats. *Acta Theriol.* **30**, 321–323 (1985).

56. Bronson, M. T. *Altidudinal Variation in the Annual Cycle and Life History of the Golden-mantled Ground Squirrel (Spermophilus Lateralis)*. (University of California, Berkeley, 1977).

57. Bartels, M. A. & Thompson, D. P. Spermophilus lateralis. *Mamm Species* 1–8 (1993) doi:10.2307/3504114.

58. Clark, T. W. Notes on the biology of the thirteen-lined ground squirrel in the Laramie Plains, Wyoming. *The Southwestern Naturalist* **15**, 499–502 (1971).

59. Streubel, D. P. & Fitzgerald, J. P. Spermophilus tridecemlineatus. *Mamm Species* 1–5 (1978) doi:10.2307/3504003.

60. Nikol’skii, A. A. Temperature conditions in burrows of the steppe marmot, Marmota bobak Müller (1776), in the hibernation period. *Russian journal of ecology* **40**, 529 (2009).

61. Braun, J. K., Eaton Jr, T. S. & Mares, M. A. Marmota caligata (Rodentia: Sciuridae). *Mammalian Species* **43**, 155–171 (2011).

62. Armitage, K. B. Social and population dynamics of yellow-bellied marmots: results from long-term research. *Annual Review of Ecology and Systematics* **22**, 379–407 (1991).

63. Frase, B. A. & Hoffmann, R. S. Marmota flaviventris. *Mamm Species* 1–8 (1980) doi:10.2307/3503965.

64. Walter, A. The evolution of marmot sociality: II. Costs and benefits of joint hibernation. *Behavioral Ecology and Sociobiology* **27**, 239–246 (1990).

65. Zervanos, S. M., Maher, C. R., Waldvogel, J. A. & Florant, G. L. Latitudinal Differences in the Hibernation Characteristics of Woodchucks (Marmota monax). *Physiological and Biochemical Zoology* **83**, 135–141 (2010).

66. Bryant, A. A. & Page, R. E. Timing and causes of mortality in the endangered Vancouver Island marmot (Marmota vancouverensis). *Canadian Journal of Zoology* **83**, 674–682 (2005).

67. Dobson, F. S. & Davis, D. E. Hibernation and Sociality in the California Ground Squirrel. *Journal of Mammalogy* **67**, 416–421 (1986).

68. Hayssen, V. Patterns of Body and Tail Length and Body Mass in Sciuridae. *J Mammal* **89**, 852–873 (2008).

69. Smith, J. E., Long, D. J., Russell, I. D., Newcomb, K. L. & Muñoz, V. D. Otospermophilus beecheyi (Rodentia: Sciuridae). *Mamm Species* **48**, 91–108 (2016).

70. Ortega, J. C. The annual cycles of activity and weight of rock squirrels (Spermophilus variegatus) in southeastern Arizona. *American Midland Naturalist* 159–171 (1991).

71. Steiner, A. L. Bedding and Nesting Material Gathering in Rock Squirrels, Spermophilus (Otospermophilus) variegatus grammurus (Say) (Sciuridae) in the Chiricahua Mountains of Arizona. *The Southwestern Naturalist* **20**, 363–369 (1975).

72. Ortega, J. C. Den Site Selection by the Rock Squirrel (Spermophilus variegatus) in Southeastern Arizona. *J Mammal* **68**, 792–798 (1987).

73. Young, P. J. Summer activity patterns of rock squirrels in central Texas. (Texas Tech University, 1979).

74. Oaks, E. C., Young, P. J., Kirkland, G. L. & Schmidt, D. F. Spermophilus variegatus. *Mamm Species* 1–8 (1987) doi:10.2307/3503949.

75. Murie, J. O. Population Characteristics and Phenology of a Franklin Ground Squirrel (Spermophilus franklinii) Colony in Alberta, Canada. *The American Midland Naturalist* **90**, 334–340 (1973).

76. Ostroff, A. C. & Finck, E. J. Spermophilus franklinii. *Mamm Species* 1–5 (2003) doi:10.1644/0.724.1.

77. Millesi, E., Strijkstra, A. M., Hoffmann, I. E., Dittami, J. P. & Daan, S. Sex and Age Differences in Mass, Morphology, and Annual Cycle in European Ground Squirrels, Spermophilus citellus. *J Mammal* **80**, 218–231 (1999).

78. Luo, J. & Fox, B. J. Life-Table Comparisons between Two Ground Squirrels. *J Mammal* **71**, 364–370 (1990).

79. Belovezhets, K. I. & Nikol’skii, A. A. Temperature regime in burrows of ground squirrels (Marmotinae) during winter hibernation. *Russ J Ecol* **43**, 155–161 (2012).

80. Broadbooks, H. E. Populations of the Yellow-Pine Chipmunk, Eutamias amoenus. *The American Midland Naturalist* **83**, 472–488 (1970).

81. Broadbooks, H. E. Life History and Ecology of the Chipmunk, Eutamias amoenus, in Eastern Washington. 56 (1958).

82. Sutton, D. A. Tamias amoenus. *Mammalian species* 1–8 (1992).

83. Broadbooks, H. E. Tree nests of chipmunks with comments on associated behavior and ecology. *Journal of Mammalogy* **55**, 630–639 (1974).

84. Bryant, M. D. Phylogeny of nearctic Sciuridae. *The American Midland Naturalist* **33**, 257–390 (1945).

85. Kawamichi, M. Nest Structure Dynamics and Seasonal Use of Nests by Siberian Chipmunks (Eutamias sibiricus). *J Mammal* **70**, 44–57 (1989).

86. Youlatos, D., Michael, D. E. & Tokalaki, K. Positional behavior of Siberian chipmunks (Tamias sibiricus) in captivity. *J Ethol* **26**, 51–60 (2008).

87. Humphries, M. M., Kramer, D. L. & Thomas, D. W. The Role of Energy Availability in Mammalian Hibernation: An Experimental Test in Free‐Ranging Eastern Chipmunks. *Physiological and Biochemical Zoology* **76**, 180–186 (2003).

88. Clarke, M. F. *et al.* Site familiarity affects escape behaviour of the eastern chipmunk, Tamias striatus. *Oikos* 533–537 (1993).

89. Pilon, L. & Baron, G. Distribution spatio-temporelle de l’activité chez Tamias striatus. *Mammalia* **54**, 391–396 (1990).

90. Essner, R. L. Morphology, locomotor behaviour and microhabitat use in North American squirrels. *J Zoology* **272**, 101–109 (2007).

91. Kenagy, G. J. & Barnes, B. M. Seasonal Reproductive Patterns in Four Coexisting Rodent Species from the Cascade Mountains, Washington. *J Mammal* **69**, 274–292 (1988).

92. Sutton, D. A. Tamias townsendii. *Mamm Species* 1–6 (1993) doi:10.2307/3504166.

93. Carey, A. B. *The biology of arboreal rodents in Douglas-fir forests*. vol. 276 (US Department of Agriculture, Forest Service, Pacific Northwest Research Station, 1992).

94. Wilk, R. J., Harrington, T. B., Gitzen, R. A. & Maguire, C. C. Forest-Floor Disturbance Reduces Chipmunk (Tamias spp.) Abundance Two Years after Variable-Retention Harvest of Pacific Northwestern Forests. *nwsc* **89**, 75–92 (2015).

95. Knopf, F. L. & Balph, D. F. Annual Periodicity of Uinta Ground Squirrels. *The Southwestern Naturalist* **22**, 213–224 (1977).

96. Slade, N. A. & Balph, D. F. Population Ecology of Uinta Ground Squirrels. *Ecology* **55**, 989–1003 (1974).

97. Eshelman, B. D. & Sonnemann, C. S. Spermophilus armatus. *Mamm Species* 1–6 (2000) doi:10.2307/0.637.1.

98. Loehr, K. A. & Risser, A. C. Daily and Seasonal Activity Patterns of the Belding Ground Squirrel in the Sierra Nevada. *Journal of Mammalogy* **58**, 445–448 (1977).

99. Zammuto, R. M. & Sherman, P. W. A comparison of time-specific and cohort-specific life tables for Belding’s ground squirrels, Spermophilus beldingi. *Can. J. Zool.* **64**, 602–605 (1986).

100. Jenkins, S. H. & Eshelman, B. D. Spermophilus beldingi. *Mamm Species* 1–8 (1984) doi:10.2307/3503911.

101. Sherman, P. W. & Runge, M. C. Demography of a Population Collapse: The Northern Idaho Ground Squirrel (spermophilus Brunneus Brunneus). *Ecology* **83**, 2816–2831 (2002).

102. Yensen, E. & Sherman, P. W. Spermophilus brunneus. *Mamm Species* 1–5 (1997) doi:10.2307/3504405.

103. Dobson, F. S., Badry, M. J. & Geddes, C. Seasonal activity and body mass of Columbian ground squirrels. *Can. J. Zool.* **70**, 1364–1368 (1992).

104. Neuhaus, P., Broussard, D. R., Murie, J. O. & Dobson, F. S. Age of Primiparity and Implications of Early Reproduction on Life History in Female Columbian Ground Squirrels. *Journal of Animal Ecology* **73**, 36–43 (2004).

105. Elliott, C. L. & Flinders, J. T. Spermophilus columbianus. *Mamm Species* 1–9 (1991) doi:10.2307/3504178.

106. Sheriff, M. J. *et al.* Phenological variation in annual timing of hibernation and breeding in nearby populations of Arctic ground squirrels. *Proceedings of the Royal Society B: Biological Sciences* **278**, 2369–2375 (2011).

107. Wilbur, S. M. Demographics and telomere dynamics of hibernating Arctic ground squirrels (Urocitellus parryii). (2019).

108. McLean, B. S. Urocitellus parryii (Rodentia: Sciuridae). *Mamm Species* **50**, 84–99 (2018).

109. Michener, G. R. The Circannual Cycle of Richardson’s Ground Squirrels in Southern Alberta. *J Mammal* **60**, 760–768 (1979).

110. Michener, G. R. Reproductive effort during gestation and lactation by Richardson’s ground squirrels. *Oecologia* **78**, 77–86 (1989).

111. Michener, G. R. & Koeppl, J. W. Spermophilus richardsonii. *Mammalian Species* 1–8 (1985).

112. Ernest, K. A. & Mares, M. A. Spermophilus tereticaudus. *Mammalian Species* 1–9 (1987).

113. Best, T. L. Spermophilus mohavensis. *Mammalian Species* 1–7 (1995).

114. Munroe, K. E. The Socioecology, Mating System and Behavior of Round-Tailed Ground Squirrels (Xerospermophilus tereticaudus). (2011).

115. Nicol, S. C. & Morrow, G. E. Sex and Seasonality: Reproduction in the Echidna (Tachyglossus aculeatus). in *Living in a Seasonal World: Thermoregulatory and Metabolic Adaptations* (eds. Ruf, T., Bieber, C., Arnold, W. & Millesi, E.) 143–153 (Springer, 2012). doi:10.1007/978-3-642-28678-0_13.

116. Levesque, D. L., Lovasoa, O. M. A., Rakotoharimalala, S. N. & Lovegrove, B. G. High mortality and annual fecundity in a free-ranging basal placental mammal, Setifer setosus (Tenrecidae: Afrosoricida). *Journal of Zoology* **291**, 205–212 (2013).

117. Stankowich, T. & Stensrud, C. Small but spiny: the evolution of antipredator defenses in Madagascar tenrecs. *J Mammal* **100**, 13–20 (2019).

118. Levesque, D. L., Rakotondravony, D. & Lovegrove, B. G. Home range and shelter site selection in the greater hedgehog tenrec in the dry deciduous forest of Western Madagascar. *Journal of Zoology* **287**, 161–168 (2012).

119. Lovegrove, B. G., Lobban, K. D. & Levesque, D. L. Mammal survival at the Cretaceous–Palaeogene boundary: metabolic homeostasis in prolonged tropical hibernation in tenrecs. *Proceedings of the Royal Society B: Biological Sciences* **281**, 20141304 (2014).

120. Nowak, R. M. *Walker’s mammals of the world: monotremes, marsupials, afrotherians, xenarthrans, and sundatherians*. (JHU Press, 2018).

121. Lohuis, T. D., Harlow, H. J., Beck, T. D. I. & Iaizzo, P. A. Hibernating Bears Conserve Muscle Strength and Maintain Fatigue Resistance. *Physiological and Biochemical Zoology* **80**, 257–269 (2007).

122. Manchi, S. & Swenson, J. E. Denning behaviour of Scandinavian brown bears Ursus arctos. *wbio* **11**, 123–132 (2005).

123. Reid, D., Jiand, M., Teng, Q., Qin, Z. & Hu, J. Ecology of the asiatic black bear (Ursus thibetanus) in Sichuan, China. *Mammalia* **55**, 221–238 (1991).

124. Seryodkin, I. V. *et al.* Denning ecology of brown bears and Asiatic black bears in the Russian Far East. *Ursus* 153–161 (2003).

125. Reid, D., Jiang, M., Teng, Q., Qin, Z. & Hu, J. Ecology of the asiatic black bear (Ursus thibetanus) in Sichuan, China. *Mammalia* **55**, 221–238 (1991).

126. Baranauskas, K. Hibernation of Barbastelle (Barbastella Barbastellas) in Šeškinė Bunkers in Vilnius (Lithuania). A Possibe Bat Population Response to Climate change. *Acta Zoologica Lituanica* **11**, 15–19 (2001).

127. Johnson, J. S., Lacki, M. J., Thomas, S. C. & Grider, J. F. Frequent arousals from winter torpor in Rafinesque’s big-eared bat (Corynorhinus rafinesquii). *PLoS One* **7**, (2012).

128. Jones, C. Plecotus rafinesquii. *Mamm Species* 1–4 (1977) doi:10.2307/3503868.

129. Beer, J. R. & Richards, A. G. Hibernation of the big brown bat. *Journal of Mammalogy* **37**, 31–41 (1956).

130. Rydell, J. Eptesicus nilssonii. *Mammalian species* 1–7 (1993).

131. Robinson, M. F. & Stebbings, R. E. Activity of the serotine bat, Eptesicus serotinus. *England. Myotis* **35**, 5–16 (1997).

132. Weller, T. J. *et al.* First direct evidence of long-distance seasonal movements and hibernation in a migratory bat. *Scientific reports* **6**, 1–7 (2016).

133. Jones, C. & Manning, R. W. Myotis austroriparius. *Mamm Species* 1–3 (1989) doi:10.2307/3504306.

134. Rice, D. W. Life history and ecology of Myotis austroriparius in Florida. *Journal of Mammalogy* **38**, 15–32 (1957).

135. Podlutsky, A. J., Khritankov, A. M., Ovodov, N. D. & Austad, S. N. A new field record for bat longevity. *The Journals of Gerontology Series A: Biological Sciences and Medical Sciences* **60**, 1366–1368 (2005).

136. Limpens, H., Lina, P. H. C. & Hutson, A. M. Action plan for the conservation of the pond bat (Myotis dasycneme) in Europe. *Council of Europe, Strasbourg* (1999).

137. Baranauskas, K. The first data about the hibernation of Daubenton’s bat (Myotis daubentonii) in the Paneriai tunnel (Vilnius, Lithuania). *Acta Zoologica Lituanica* **13**, 379–384 (2003).

138. Decher, J. & Choate, J. R. Myotis grisescens. *Mammalian Species* 1–7 (1995).

139. Caire, W., LaVal, R. K., LaVal, M. L. & Clawson, R. Notes on the ecology of Myotis keenii (Chiroptera, Vespertilionidae) in eastern Missouri. *American Midland Naturalist* 404–407 (1979).

140. Norquay, K. J. O. & Willis, C. K. R. Hibernation phenology of M yotis lucifugus. *Journal of Zoology* **294**, 85–92 (2014).

141. Zahn, A., Rodrigues, L., Rainho, A. & Palmeirim, J. M. Critical times of the year for Myotis myotis, a temperate zone bat: roles of climate and food resources. *Acta Chiropterologica* **9**, 115–125 (2007).

142. Kaupas, L. Roosting Behaviour and Thermoregulation of the Northern Long-Eared Bat (Myotis septentrionalis) Near the Northern Extent of its Range. (Graduate Studies, 2016).

143. Wilkinson, G. S. & South, J. M. Life history, ecology and longevity in bats. *Aging Cell* **1**, 124–131 (2002).

144. Kurta, A. *Mammals of the Great Lakes Region.* (The University of Michigan, 1995).

145. Caceres, M. C. & Barclay, R. M. R. Myotis septentrionalis. *Mamm Species* 1–4 (2000) doi:10.2307/0.634.1.

146. Clawson, R. L., LaVal, R. K., LaVal, M. L. & Caire, W. Clustering behavior of hibernating Myotis sodalis in Missouri. *Journal of Mammalogy* **61**, 245–253 (1980).

147. Caire, W. & Loucks, L. S. Loss in mass by hibernating Cave Myotis, Myotis velifer (Chiroptera: Vespertilionidae) in western Oklahoma. *The Southwestern Naturalist* **55**, 323–330 (2010).

148. J.w, S. & P.f, V. H. Seasonal Habits of the Noctule Bat (Nyctalus Noctula). *Archives Néerlandaises de Zoologie* **16**, 423–439 (1964).

149. Dalhoumi, R., Morellet, N., Aissa, P. & Aulagnier, S. Seasonal activity pattern and habitat use by the Kuhl’s pipistrelle (Pipistrellus kuhlii) in an arid environment. *European journal of wildlife research* **64**, 36 (2018).

150. Racey, P. A. Ageing and assessment of reproductive status of pipistrelle bats, Pipistrellus pipistrellus. *Journal of Zoology* **173**, 264–271 (1974).

151. Damm, J. P. & Geluso, K. Use of a mine by eastern pipistrelles (Perimyotis subflavus) in east central Nebraska. *Western North American Naturalist* **68**, 382–389 (2008).

152. Stebbings, R. E. A Comparative Study of Plecotus Auritus and P. Austriacus (Chiroptera, Vespertilionidae) Inhabiting One Roost. *Bijdragen tot de Dierkunde* **40**, 91–94 (1970).

153. Herrero, S. Aspects of evolution and adaptation in American black bears (Ursus americanus Pallas) and brown and grizzly bears (U. arctos Linne.) of North America. *Bears: Their biology and management* 221–231 (1972).
