# Supplementary material for: Integrating Mortality Risk and the Adaptiveness of Hibernation
Source: Front Physiol. 2020 Jul 10;11:706. doi: 10.3389/fphys.2020.00706 (PMC7366871; doi:10.3389/fphys.2020.00706)
Supplement: Supplementary file 5 [file Table_3.docx]

|  |  |  | λ_ML_ | |
| --- | --- | --- | --- | --- |
|  |  | **Model terms** | **AIC** | **R^2^ / R^2^_adj_** |
| Phylogenetic correction  (PGLS) | Model 1:  All hibernators  (82 species) | zhiber+zlogmass+zhiber:zlogmass+C_W+bats+arboreality | -50.51046 | 0.2653/ 0.2065 |
|  |  | zhiber+zlogmass+C_W+bats+arboreality | -52.49145 | 0.2637/0.2152 |
|  |  | zhiber+zlogmass+bats+arboreality | -52.01818 | 0.2242/0.1839 |
|  | Model 2:  Hibernators without bats  (55 species) | zhiber+zlogmass+zhiber:zlogmass+C_W+arboreality | -42.84827 | 0.4048/0.3441 |
|  |  | zhiber+zlogmass+C_W+arboreality | -44.84158 | 0.4038/0.3561 |
|  |  | zhiber+zlogmass+arboreality | -44.92069 | 0.3744/0.3376 |
|  | Model 3:  Deep hibernators without bats (46 species) | zhiber+zlogmass+zhiber:zlogmass+C_W+arboreality  zhiber+zlogmass+C_W+arboreality  zhiber+zlogmass+arboreality  zhiber+zlogmass+ | -37.18019  -39.17963  -38.92486  -40.34324 | 0.2105/0.1118  0.2104/0.1334  0.1718/0.1126  0.1592/0.1201 |
|  | Model 4:  Small hibernators (<1,5 kg) without bats (44 species) | zhiber+zlogmass+zhiber:zlogmass+C_W+arboreality | -48.57117 | 0.3206/0.2312 |
|  |  | zhiber+zlogmass+C_W+arboreality | -49.04655 | 0.3038/0.2324 |
|  |  | zhiber+zlogmass+arboreality | -48.3023 | 0.2662/0.2112 |
| No phylogenetic correction  (linear model) | Model 5:  Bats only (27 species) | zhiber+zlogmass+zhiber:zlogmass | -7.308115 | 0.193/0.0877 |
|  |  | zhiber+zlogmass | -8.912492 | 0.1811/0.1128 |

Table S3: Model selection for the effects of hibernation season duration (zhiber), body mass (zlogmass), the interaction zhiber x zlogmass, bats and arboreality lifestyle and the effect of captivity (C_W; captive vs. wild) on longevity in hibernating mammals.
